# Mechanism for the reactivation of the peroxidase activity of human cyclooxygenases: investigation using phenol as a reducing cosubstrate

**DOI:** 10.1038/s41598-020-71237-x

**Published:** 2020-09-16

**Authors:** Chengxi Yang, Peng Li, Xiaoli Ding, Hao Chen Sui, Shun Rao, Chia-Hsiang Hsu, Wing-Por Leung, Gui-Juan Cheng, Pan Wang, Bao Ting Zhu

**Affiliations:** 1 Shenzhen Key Laboratory of Steroid Drug Discovery and Development, The Chinese University of Hong Kong, Shenzhen, 518172 China; 2 School of Life and Health Sciences, The Chinese University of Hong Kong, 2001 Longxiang Road, Longgang District, Shenzhen, 518172 China; 3 School of Science and Engineering, The Chinese University of Hong Kong, Shenzhen, 518172 China; 4 Shenzhen Bay Laboratory, Shenzhen, 518055 China

**Keywords:** Computational chemistry, Enzyme mechanisms, Pharmacology

## Abstract

It has been known for many years that the peroxidase activity of cyclooxygenase 1 and 2 (COX-1 and COX-2) can be reactivated in vitro by the presence of phenol, which serves as a reducing compound, but the underlying mechanism is still poorly understood. In the present study, we use phenol as a model compound to investigate the mechanism by which the peroxidase activity of human COXs is reactivated after each catalytic cycle. Molecular docking and quantum mechanics calculations are carried out to probe the interaction of phenol with the peroxidase site of COXs and the reactivation mechanism. It is found that the oxygen atom associated with the Fe ion in the heme group (i.e., the complex of Fe ion and porphyrin) of COXs can be removed by addition of two protons. Following its removal, phenol can readily bind inside the peroxidase active sites of the COX enzymes, and directly interact with Fe in heme to facilitate electron transfer from phenol to heme. This investigation provides theoretical evidence for several intermediates formed in the COX peroxidase reactivation cycle, thereby unveiling mechanistic details that would aid in future rational design of drugs that target the peroxidase site.

## Introduction

Cyclooxygenase 1 and 2 (COX-1 and COX-2) are key enzymes involved in the formation of many essential bio-mediators, including prostaglandins (PGs), hydroxyeicosateraenoic acids (HETEs), and thromboxanes, etc^1–4^. While COX-1 is a house-keeping enzyme that is expressed in most tissues, COX-2 works as an inducible enzyme mediating pathological conditions such as inflammation, and is an important target for anti-inflammatory drugs, like nonsteroidal anti-inflammatory drugs (NSAIDs)^5^.

The COX-1 and COX-2 enzymes have two functionally-coupled active sites: the cyclooxygenase site that converts arachidonic acid (AA) to prostaglandin G_2_ (PGG_2_), and the peroxidase site that catalyzes the reaction which reduces PGG_2_ to prostaglandin H_2_ (PGH_2_). These two catalytic activities are linked in a proposed branched model^6^, in which hydroperoxide is required to initiate the catalytic cycle by oxidizing the resting heme group (i.e., the complex of Fe ion and (proto)porphyrin) to protoporphyrin IX (PPIX) radical cation with an oxyferryl group (Fe^IV^=O), known as *Compound I*. Next, *Compound I* extracts one electron from Tyr385 and becomes *Compound II*, i.e., a neutral PPIX with an oxyferryl group. The Tyr385 radical formed initiates the non-stopping cyclooxygenase cycle, which produces PGG_2_ until suicide inactivation takes place, and *Compound II* is subsequently reduced to the resting state by cosubstrates. After the first round of initiation, the resting heme is oxidized by PGG_2_ to *Compound I* which is then reduced by cosubstrates instead of Tyr385, thereby allowing the catalytic cycle to continually convert PGG_2_ to PGH_2_^6–8^.

During the in vitro enzymatic assay of COX-mediated reactions, phenol is usually included, which was found many years ago to be capable of serving as an activator of the COX peroxidase activity^9,10^, but the mechanism of its action is still unclear. Our recent studies have demonstrated that certain natural flavonoids can activate COX as reducing cosubstrates, which accelerate the conversion of *Compound I* to its resting state^11,12^. These observations have led to the hypothesis that phenol might stimulate COX enzymes via a similar mechanism like flavonoids, for they all have reducing hydroxyl groups attached to an aromatic ring. Besides, phenol resembles the side chain of Tyr385, which reduces *Compound I* at the initiation phase of the cyclooxygenase cycle.

**Scheme 1:**
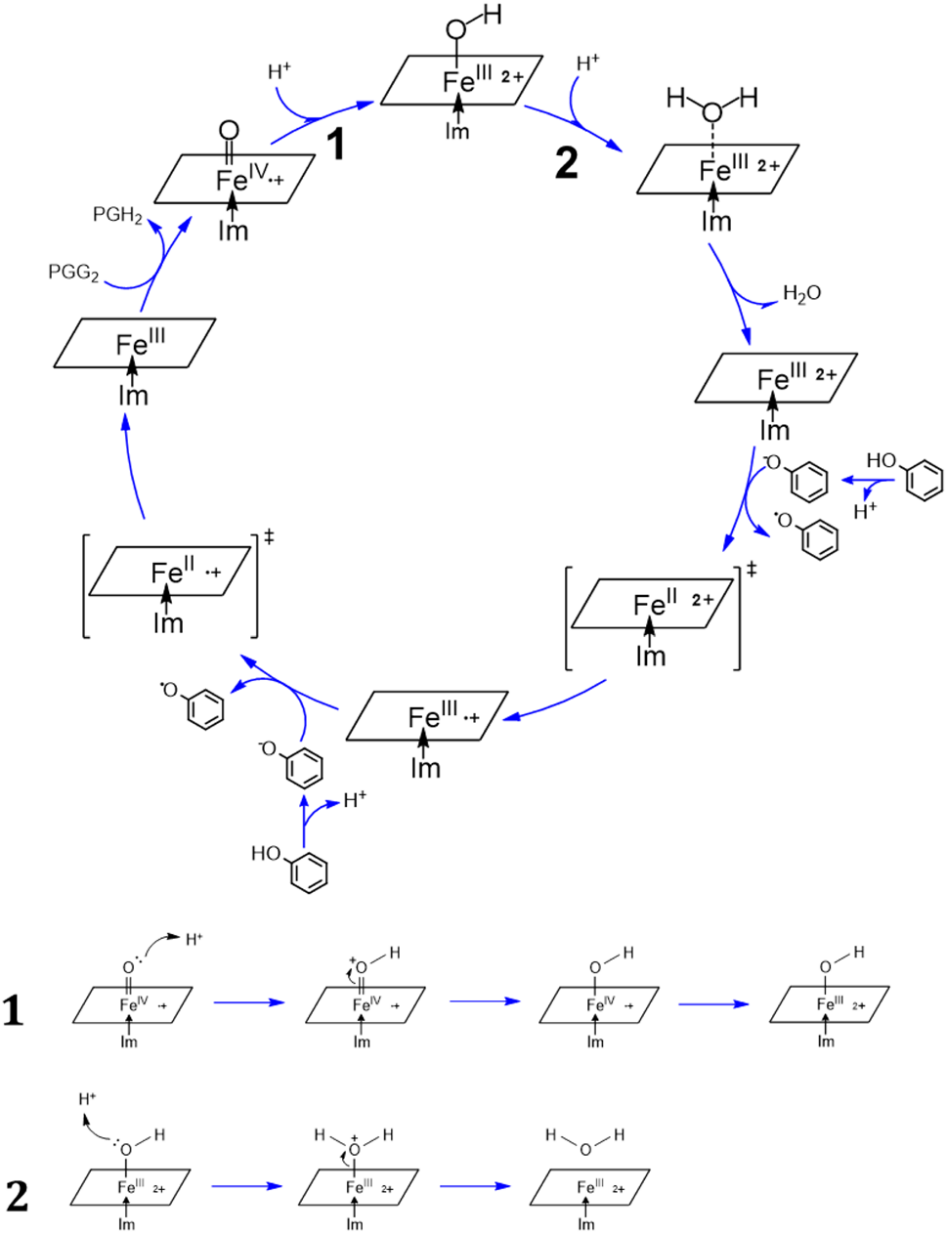
A proposed mechanism for the reactivation of the catalytic cycle of the COX peroxidase by phenol. PPIX is shown as an abbreviated parallelogram, and the imidazole ring of His388 as Im. Also, PGG_2_ is for prostaglandin G_2_, and PGH_2_ for prostaglandin H_2_. The proposed mechanisms for reaction 1 and 2 are depicted beneath the main scheme. In these two reactions, two protons attack the oxygen atom of Fe=O, resulting in the formation of H_2_O molecule. Detailed molecular orbital analysis of reaction 1 is shown in Scheme 2.

In this investigation, we jointly use molecular docking approach and quantum chemistry calculations to study the binding interaction of phenol with COX-1 and COX-2 and how it activates their COX peroxidase activity. We find that phenol can bind inside the COX’s peroxidase active site after removal of the oxygen atom associated with the heme’s Fe ion, and then phenol directly interacts with heme to facilitate its electron transfer to heme (Scheme 1). The finding of this study provides a mechanistic understanding and structural basis for phenol to function as a reducing cosubstrate of COX-1 and COX-2.

## Methods

### Molecular docking

Energy minimization and molecular docking are performed with the *Discovery Studio* modeling software (Version 16.1.0.15350, Dassault Systèmes BIOVIA, San Diego, CA, U.S.A.) installed in a Windows Server R2 operating system on a Dell PowerEdge R730 Server^13^.

### Protein structure refinement

The X-ray structure of human COX-1 (PDB code: 6y3c^14^) and human COX-2 (PDB code: 5kir^15^) are used as templates for computational docking analyses. For comparison, the X-ray structure of sheep COX-1 protein (PDB code: 1q4g^16^) and mouse COX-2 protein (PDB code: 3nt1^17^) in complex with protoporphyrin IX (PPIX) containing an Fe^III^ ion (abbreviated as PPIXFe^III^) are also included in the computational docking analysis. All small molecules that are non-covalently attached to the COX proteins are removed, and then the amino acid residues in the protein structures are renumbered according to the known sequences. The PPIXFe^III^ is individually add to the peroxidase sites of human COX-1 and COX-2. The *Clean Protein* module in *Discovery Studio* is used to complete the side chains for amino acid residues, to correct bonding and bond orders, and to add hydrogens back. Notably, the PPIXFe^III^ component in both COX-1 and COX-2 are modified into PPIX^•+^Fe^IV^=O, PPIX^2+^Fe^III^ and PPIX^+^Fe^III^ for further docking analysis. The reason for using PPIX^•+^Fe^IV^ = O, PPIX^2+^Fe^III^ and PPIX^•+^Fe^III^ in place of PPIXFe^III^ is described later in the Results section. The *Prepare Protein* module in *Discovery Studio* is used together with the *CHARMm* force field for protein preparation^18^.

### Flexible docking

For flexible docking, we use the *Find Sites from Receptor Cavities* module to identify the binding site in the prepared COX-1 and COX-2 structures. According to our earlier study, the target site is the peroxidase active site in these two COX proteins^12^. We select all amino acid residues within a 5 Å reach of the target site and allow them to have flexible side chains. The *SBD Site Sphere* is centered at the target site and then expanded to the radius size around 13 Å. Under the *Flexible Docking* mode with the maximum number of residues for creating side chain conformations set to 10, the *Simulated Annealing* docking method is then applied to dock phenol into the target site of COX-1 and COX-2. Docking analyses are separately carried out for COX-1 and COX-2, with phenol in both ionized and nonionized states. The whole structure of each COX protein is further minimized with the *CHARMm* force field^18^. This docking method is validated by performing a “self-docking” exercise using the crystal structure of mouse COX-2 in complex with celecoxib (PDB id: 3ln1)^19^. Specifically, we remove celecoxib from the experimentally-determined structure of the complex, and then use the computational docking program to dock it back inside the COX-2 protein. The root mean square of deviation (RMSD) between the docked ligand pose and its experimentally-determined pose in the crystal structure is computed, which is found to be 0.63 Å, suggesting that the docking method employed in this study has a very good ability to predict the correct binding pose(s) of a small ligand inside the specified COX binding pocket (Figure S1).

### Calculation of binding energy

After completing flexible docking, the *Calculate Binding Energies* module is used to find complexes with the lowest binding energy value. The free energy of binding for a receptor-ligand complex is computed from the free energies of the complex, the target protein, and the ligand. According to *Discovery Studio*, the free energy values are separately computed using the *CHARMm* force field and the Poisson-Boltzmann equation with the non-polar surface area (PBSA) method^18,20^. In this approach, the Poisson–Boltzmann equation is solved numerically on a three-dimensional (3D) grip, and the computed electrostatic potential is used to estimate the electrostatic solvation free energy. The ligand conformational entropy is also taken into consideration during the free binding energy calculation. The following equation is used to calculate the binding energy (Δ*G*_binding_) between phenol and the COX-1 or COX-2 protein:

$${{\varvec{\Delta}}}G_{{{\mathbf{binding}}}} \, = \,G_{{{\mathbf{complex}}}} - \left( {G_{{{\mathbf{COX}}}} \, + \,G_{{{\mathbf{ligand}}}} } \right)$$

where *G*_complex_ is the absolute free energy of the complex, *G*_COX_ is the absolute free energy of the COX protein, and *G*_ligand_ is the absolute free energy of the ligand^21,22^. The Δ*G*_binding_ value is used to reflect the relative interaction affinity between the COX enzyme and the phenol molecule.

### Quantum chemistry calculation of geometry and Gibbs free energy

The molecular geometry optimization and vibrational frequency calculation are performed on a Dell PowerEdge R730 Server with *Gaussian* 09 W calculation software (Revision D.01, Gaussian, Wallingford, CT)^23^. Geometrical structure and electronic information of molecules are investigated in the vapor phase using the B3LYP-D3 method (namely, the Becke’s three-parameter hybrid functional and the Lee–Yang–Parr correlation functional method)^24–28^. The 6–311+G(d,p) basis set is used for C, H, N and O^29–32^. The def2-TZVP basis set is used for Fe^33,34^. The structures of the molecules of interest are optimized first, and the optimized geometries are then undergone vibrational frequency calculation. The calculated vibrational frequency values are used to aid in verifying the stationary points to be the real minimal values and also in obtaining thermal corrections at 310.00 K. These computed values would help reflect the stability of the optimized structures.

The quantum theory of atoms in molecular analysis is implemented in the *Multiwfn* software (version 3.7)^35^ via analysis of the wave function obtained from the *Gaussian* optimization. Mayer bond order is a natural extension of the Wiberg bond order on a nonorthogonal basis. In general, Mayer bond order is in good agreement with the empirical bond order; for the classical single, double, and triple bonds, their values are close to 1.0, 2.0, and 3.0, respectively. Atomic charge is described according to the Mulliken method^36^.

### Qualitative molecular orbital analyses

To pair the α and β orbitals generated by unrestricted open shell calculations, biorthogonalization is performed on basis of the canonical orbitals which are obtained from the single point calculation of optimized structures using the *Multiwfn* software^35^. The overlap integral matrix ${\varvec{O}}$ between alpha and beta orbitals is constructed first, and then decomposed by Single Value Decomposition (SVD) as:

$${\varvec{O}}={\varvec{U}}\boldsymbol{\Sigma }{\varvec{V}}$$

where $\boldsymbol{\Sigma }$ is a diagonal matrix, whose diagonal values correspond to the overlap integrals between the newly transformed alpha and beta orbitals. Normally, such new overlap integrals should approximate 1.0. The parameter ***U ***(***V***) is the transformation matrix between the original orbitals and the new orbitals of α (β) spin^37^. The coefficient matrix of the biorthogonalized orbitals can be calculated as:

$${{\varvec{C}}}_{{\varvec{b}}{\varvec{i}}{\varvec{o}}{\varvec{r}}{\varvec{t}}{\varvec{h}}{\varvec{o}}}^{\boldsymbol{\alpha }}={\varvec{U}}{{\varvec{C}}}_{{\varvec{o}}{\varvec{r}}{\varvec{i}}{\varvec{g}}{\varvec{i}}{\varvec{n}}{\varvec{a}}{\varvec{l}}}^{\boldsymbol{\alpha }}$$

$${{\varvec{C}}}_{{\varvec{b}}{\varvec{i}}{\varvec{o}}{\varvec{r}}{\varvec{t}}{\varvec{h}}{\varvec{o}}}^{{\varvec{\beta}}}={\varvec{V}}{{\varvec{C}}}_{{\varvec{o}}{\varvec{r}}{\varvec{i}}{\varvec{g}}{\varvec{i}}{\varvec{n}}{\varvec{a}}{\varvec{l}}}^{{\varvec{\beta}}}$$

The energy of biorthogonalized orbitals is evaluated as the expectation value of Fock operator:

$${{\varvec{F}}}_{{\varvec{b}}{\varvec{i}}{\varvec{o}}{\varvec{r}}{\varvec{t}}{\varvec{h}}{\varvec{o}}}={{\varvec{C}}}^{{\varvec{T}}}{{\varvec{F}}}_{{\varvec{A}}{\varvec{O}}}{\varvec{C}}$$

where ${{\varvec{F}}}_{{\varvec{A}}{\varvec{O}}}$ is the Fock matrix in original basis function, and ${\varvec{C}}({\varvec{\mu}},{\varvec{i}})$ corresponds to the coefficient of basis function ${\varvec{\mu}}$ in biorthogonalized orbital ${\varvec{i}}$. After energy calculation, biorthogonalized orbitals are ranked according to their energies (average of energy of α orbital and its β counterpart)^37^.

## Results and discussion

**Figure 1:**
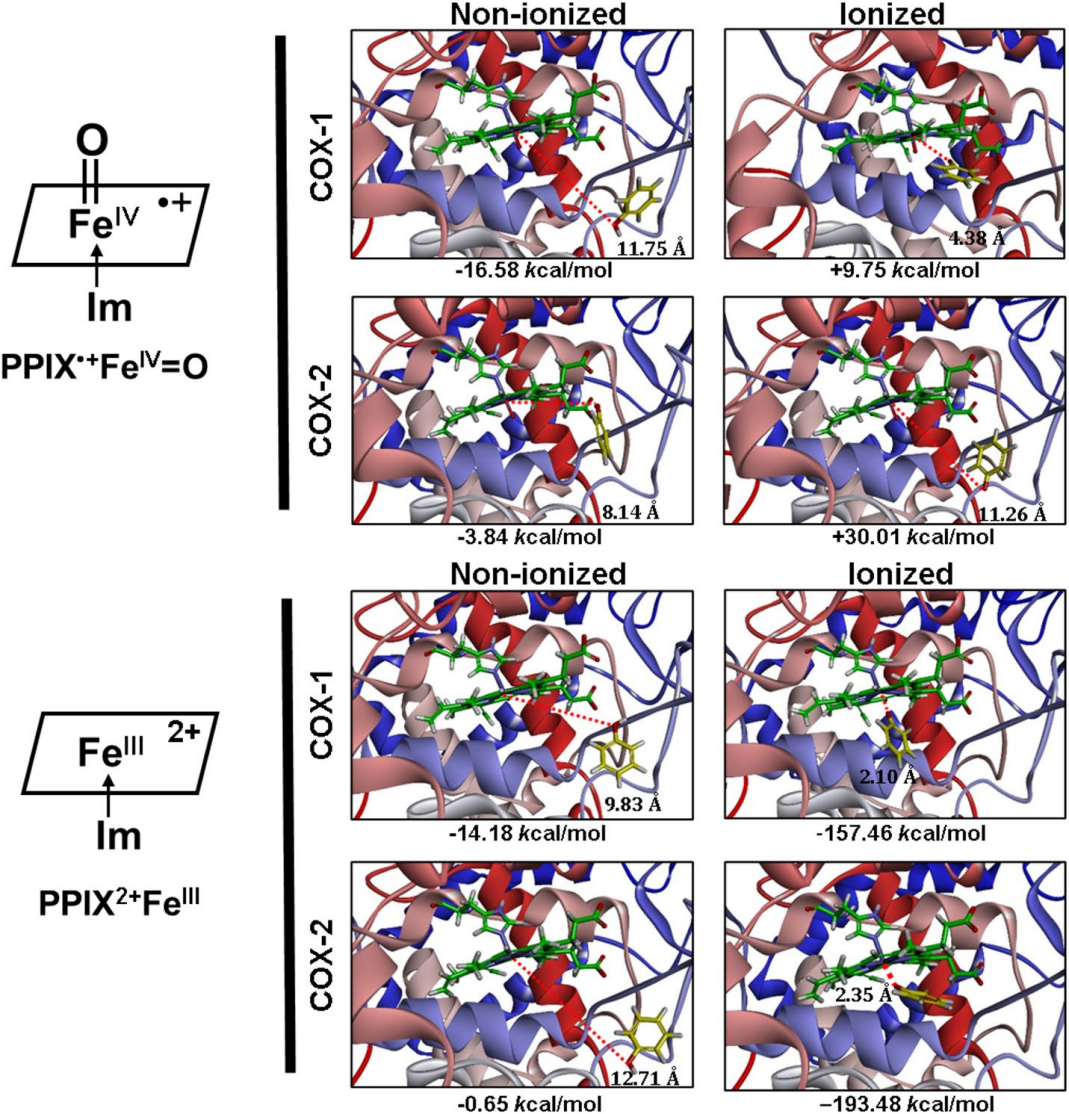
Molecular docking analysis of the binding mode of phenol in both non-ionized and ionized states inside the peroxidase active sites of human COX-1 and COX-2 with PPIX^•+^Fe^IV^=O and PPIX^2+^Fe^III^. The protein structures are shown as solid ribbons. Carbon atoms in PPIX^•+^Fe^IV^=O and PPIX^2+^Fe^III^ are colored in green, nitrogen in blue, oxygen in red, hydrogen in white, and iron in bice. Carbon atoms in phenol are colored in yellow, oxygen in red and hydrogen in white. In addition, the dash line corresponds to the distance between phenol’s oxygen atom and iron in PPIX^•+^Fe^IV^=O or PPIX^2+^Fe^III^.

First, the *Flexible Docking* function of *Discovery Studio* is used to dock the phenol molecule in both non-ionized and ionized states into the peroxidase active site with an oxygen atom covalently attached to the Fe^IV^ ion (abbreviated as Fe^IV^=O) of COX-1 and COX-2 to predict the potential binding mode and orientation of phenol’s O atom toward the Fe atom in PPIX^•+^Fe^IV^=O. Results from our recent studies^38,39^ suggest that the binding interaction of a reducing substrate under ionization is dramatically enhanced in comparison with the non-ionizing state. Therefore, in the present study, we also choose to perform docking analysis using both non-ionized and ionized phenol molecules. Under these conditions, we find that either the binding energies between phenol molecule (non-ionized or ionized) and PPIX^•+^Fe^IV^=O are positive, or the distances between phenol’s oxygen and the Fe^IV^ ion are longer than 5 Å, which indicate that the presence of the Fe=O group in PPIX^•+^Fe^IV^=O would prevent the binding of phenol or phenol ion inside the peroxidase active site close to the heme group (Fig. 1, Table 1).

**Table 1:** Binding energies and Fe–O distances of non-ionized and ionized phenol molecules docked inside the COX-1 and COX-2 peroxidase sites.

State of the peroxidase site	Binding energy (kcal/mol)		Fe‒O distance (Å)	
	Non-ionized	Ionized	Non-ionized	Ionized
COX-1 PPIX^•+^Fe^IV^=O	‒16.58	+ 9.75	11.75	4.38
COX-2 PPIX^•+^Fe^IV^=O	‒3.84	+ 30.01	8.14	11.26
COX-1 PPIX^2+^Fe^III^	‒14.18	‒157.46	9.83	2.10
COX-2 PPIX^2+^Fe^III^	‒0.65	‒193.48	12.71	2.35
COX-1 PPIX^+^Fe^III^	‒15.84	‒100.71	12.06	2.11
COX-2 PPIX^+^Fe^III^	‒7.13	‒139.32	11.94	2.11

Next we further test the possibility of whether phenol in its non-ionized or ionized state can be docked inside the peroxidase active sites of COX-1 and COX-2 when the O atom is absent in the Fe=O group (namely, in the form of PPIX^2+^Fe^III^). We find that both ionized and non-ionized phenol molecules can be docked inside the active site of COX-1 and COX-2 (Fig. 1, Table 1). As expected, ionized phenol has a much lower binding energy level and is much closer to Fe than non-ionized phenol, indicating that the former can bind more favorably inside the peroxidase sites of COX-1 and COX-2 than the latter. The binding energies of phenol ion are ‒157.46 kcal/mol for COX-1 and ‒193.48 kcal/mol for COX-2, respectively. As is shown in Fig. 1, the oxygen atom of phenol ion is very close to the heme Fe when O is removed from the Fe=O group. The Fe‒O distances of the docked phenol ion are 2.10 Å for COX-1 and 2.35 Å for COX-2, which is sufficiently close for electron transfer to take place. Similar results are also obtained using sheep COX-1 and mouse COX-2 as target proteins (data are summarized in Figure S2). The high degree of similarity in their docking results is not surprising, as these proteins share a high degree of sequence homology (89% for human and sheep COX-1 proteins and 85% for human and mouse COX-2 proteins).

This result leads to the suggestion that the binding of phenol inside the peroxidase active sites of COX-1 and COX-2 likely occurs only after the O atom is removed from Fe^IV^=O. Regarding the possible mechanism of oxygen removal from Fe^IV^=O, we hypothesize that two protons might bind to the oxygen atom in PPIX^•+^Fe^IV^=O and then the oxygen atom is removed by forming H_2_O (depicted in Scheme 1). To test this hypothesis, we conducted a theoretical analysis of the binding process of two protons to the O atom in PPIX^•+^Fe^IV^=O. Since these calculations are extremely time-consuming, here we adopt a slightly simplified approach by using Por^•+^Fe^IV^=O (Por^•+^ refers to porphyrin π radical) as a model compound for *Gaussian* calculation. In this model, the His388, which binds to the heme iron in COX enzymes, is simplified into an imidazole ring (abbreviated as Im). Notably, this simplified model has also been used in earlier studies on cytochrome P450 enzymes^40–42^.

Before examining our proposed cycle, we first calculate the electronic structures including spin density (Table 2, Table S1 and S2) and molecular orbitals of all intermediates (^2^Por^•+^Fe^IV^=O and ^4^Por^•+^Fe^IV^=O are shown as examples in Scheme S1 and Figure S3). Molecular orbital analyses indicate that for either doublet or quartet state, there are 3 unpaired electrons located in π^*^ (d_yz_-p_y_), π^*^ (d_xz_-p_x_), and a_2u_ orbitals. This result is consistent with the spin density result (Table 2). The sum of the spin densities of iron and oxygen atoms is approximately 2.0, which corresponds to the two singly-occupied orbitals π^*^ (d_yz_-p_y_) and π^*^ (d_xz_-p_x_). The spin density of porphyrin is approximately 1.0 (or ‒1.0), which corresponds to the singly-occupied a_2u_ orbital of the porphyrin aromatic ring (Scheme S1 and 2). Such electronic structures (doublet and quartet states) match well with the results from previous studies of cytochrome P450, a protein with a similar heme structure in the active site^40–42^.

**Table 2:** Relative energies (kcal/mol), group spin densities (ρ), and NBO charge (q) of optimized structures.

Name	Relative energy^a^	ρ_Fe_	ρ_Porphrin_	ρ_O_^b^	ρ_Imidazole_	q_Fe_	q_Porphrin_	q_O_^c^	q_Imidazole_
^2^Por^•+^Fe^IV^=O	0.00	1.1	‒1.0	0.9	0.0	0.3	0.7	‒0.3	0.3
^4^Por^•+^Fe^IV^=O	‒0.88	1.0	1.0	1.0	0.0	0.3	0.7	‒0.3	0.3
^6^Por^•+^Fe^IV^=O	11.67	2.8	1.4	0.8	0.0	0.3	0.8	‒0.4	0.3
^2^Por^2+^Fe^III^‒OH	‒29.70	0.9	0.0	0.1	0.0	0.3	1.6	‒0.3	0.4
^4^Por^2+^Fe^III^‒OH	‒25.16	0.8	2.0	0.2	0.0	0.3	1.6	0.3	0.4
^6^Por^2+^Fe^III^‒OH	‒18.13	2.4	2.0	0.4	0.2	0.7	1.5	‒0.4	0.2
^2^Por^2+^Fe^III^‒OH_2_	‒54.77	1.0	0.0	0.0	0.0	0.5	1.6	0.0	0.0
^4^Por^2+^Fe^III^‒OH_2_	‒53.69	1.0	2.0	0.0	0.0	0.4	1.8	0.5	0.3
^6^Por^2+^Fe^III^‒OH_2_	‒55.18	2.6	2.2	0.0	0.2	0.8	1.8	0.2	0.2
^2^Por^2+^Fe^III^	‒55.60	1.0	0.0	‒	0.0	0.7	1.7	‒	0.6
^4^Por^2+^Fe^III^	‒45.65	2.6	0.2	‒	0.2	1.0	1.7	‒	0.3
^6^Por^2+^Fe^III^	‒57.05	2.7	2.1	‒	0.2	1.0	1.6	‒	0.4
^1^Por^2+^Fe^II^	‒82.42	0.0	0.0	‒	0.0	0.5	1.1	‒	0.4
^3^Por^•+^Fe^III^	‒102.55	1.0	1.0	‒	0.0	0.7	0.8	‒	0.5
^5^Por^•+^Fe^III^	‒107.63	2.7	1.1	‒	0.2	1.0	0.7	‒	0.3
^7^Por^•+^Fe^III^	‒103.46	4.0	1.8	‒	0.2	1.3	0.4	‒	0.3
^2^PorFe^III^	‒131.60	1.0	0.0	‒	0.0	0.7	‒0.2	‒	0.5
^4^PorFe^III^	‒142.23	2.7	0.1	‒	0.2	0.9	‒0.2	‒	0.3
^6^PorFe^III^	‒135.71	4.0	0.9	‒	0.1	1.3	‒0.6	‒	0.3

^a^Sum of the whole complex

^b^Spin density of O atom, OH group, and OH_2_ group. ^c^NBO charge of O atom, OH group, and OH_2_ group.

**Figure 2:**
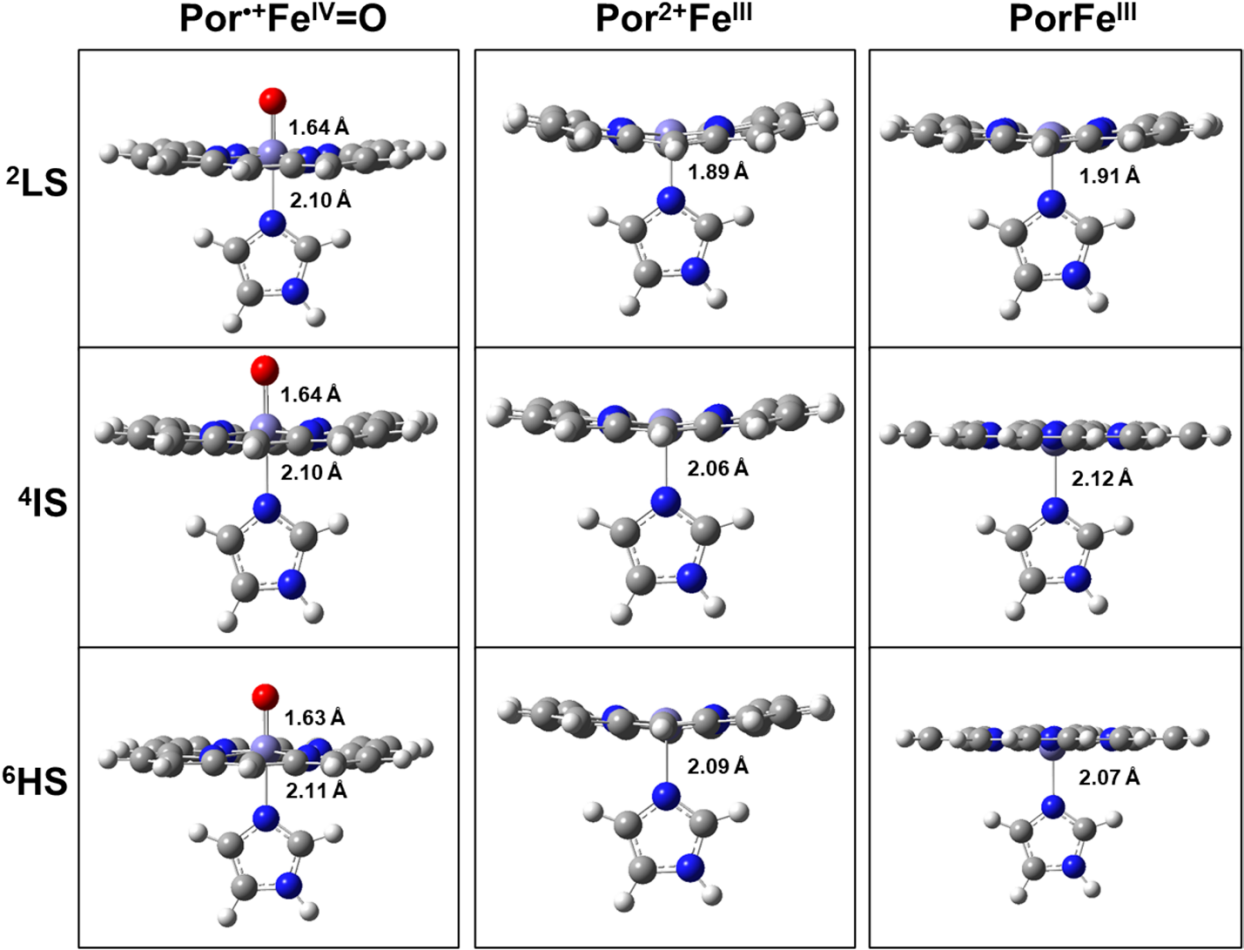
Optimization of reaction between two protons and Por^•+^Fe^IV^=O. ^*2*^*LS* doublet spin state, ^*4*^*IS* quartet spin state, ^*6*^*HS* sextet spin state. Color representation of atoms: gray for carbon, white for hydrogen, red for oxygen, and blue for nitrogen.

Optimized geometries of the complexes Por^•+^Fe^IV^=O, Por^2+^Fe^III^ and PorFe^III^ in the lowest doublet, quartet and sextet spin states are schematically depicted in Fig. 2. All three electronic states in Fig. 2 correspond to the minimum energy structures that have zero imaginary frequencies. In terms of geometry, the doublet and quartet states of Por^•+^Fe^IV^=O closely resemble each other. In addition, the quartet and sextet states of Por^2+^Fe^III^ are also very similar. In the case of the PorFe^III^ structures, the epoxide moiety is in a plane almost orthogonal to the porphyrin ring bisecting the plane between the four nitrogen atoms. In particular, the quartet state has a substantially shorter Fe-imidazole bond length of 2.10 Å in ^4^Por^•+^Fe^IV^=O compared to 2.06 Å in ^2^Por^2+^Fe^III^.

**Figure 3:**
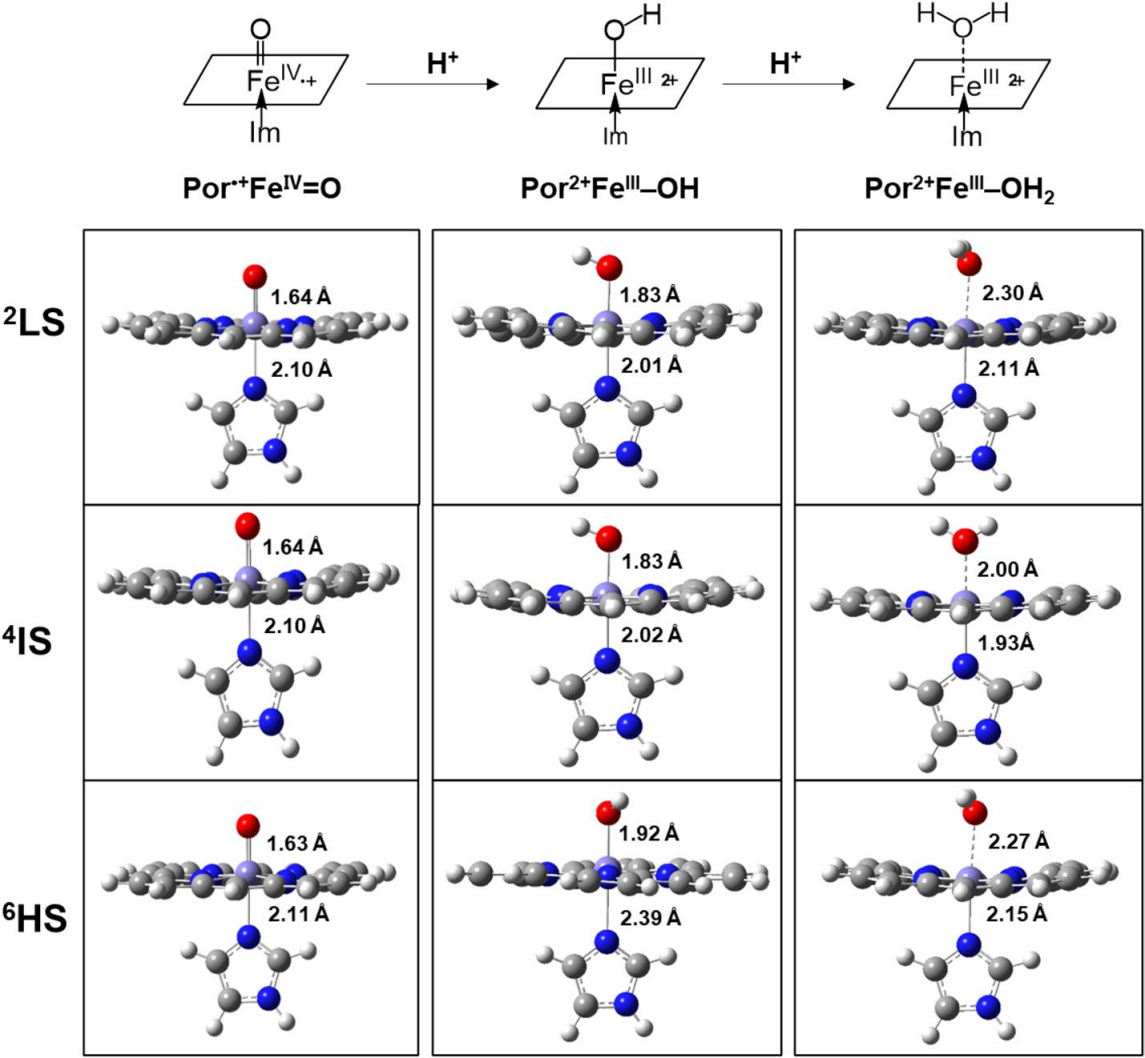
Optimized structures of Por^•+^Fe^IV^=O, Por^2+^Fe^III^ and PorFe^III^. ^2^LS doublet spin state, ^4^IS quartet spin state, ^6^HS sextet spin state. Color representation of atoms: gray for carbon, white for hydrogen, red for oxygen, and blue for nitrogen.

**Scheme 2:**
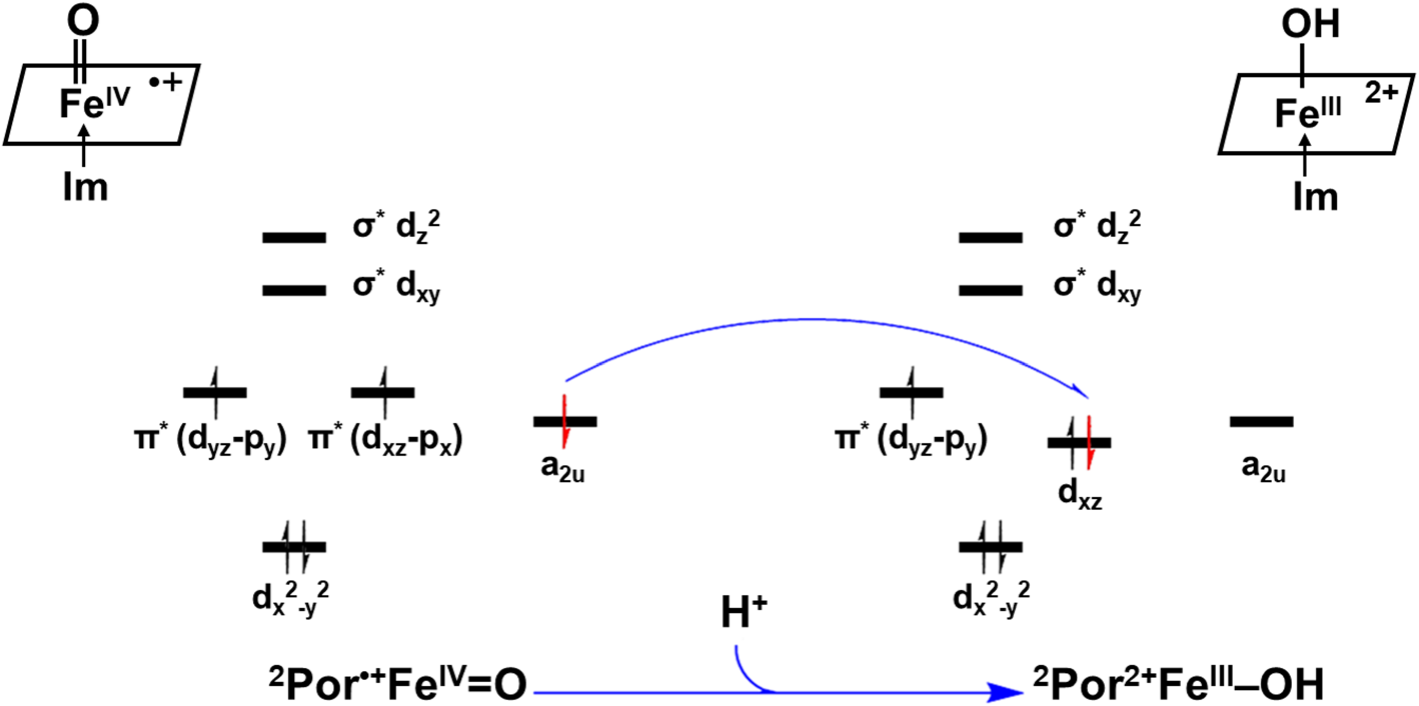
Electron transfer from ^2^Por^•+^Fe^IV^=O to ^2^Por^2+^Fe^III^‒OH when the first proton binds to the oxygen atom of the Fe=O group.

The reaction of the first proton binding to Por^•+^Fe^IV^=O is depicted in the lower panel of Scheme 1 as reaction **1**. This mechanism is supported by our quantum mechanics calculations as follows. As the first proton binds to the O atom in ^2^Por^•+^Fe^IV^=O (Fig. 3), the bond length of Fe^IV^‒O is slightly increased from 1.64 to 1.83 Å (Table 3), indicating that its binding to ^2^Por^•+^Fe^IV^=O weakens Fe‒O bond strength. Meanwhile, the Mayer bond order of Fe‒O in Por^2+^Fe^III^‒O decreases from 1.47 to 0.82 (Table 3), which also reflects the weakening of the Fe‒O bond following the bonding of the first proton. Similarly, ^4^Por^•+^Fe^IV^=O and ^6^Por^•+^Fe^IV^=O show the same trend in these states. Moreover, the spin density analysis suggests that the binding of the first proton changes the electronic structure of iron and porphyrin (Table 2). From ^2^Por^•+^Fe^IV^=O to ^2^Por^2+^Fe^III^‒OH, the spin density of the porphyrin changes from − 1.0 to 0.0, and its NBO charge increases from 0.7 to 1.6 (Table 2). This indicates that the original single electron on the a_2u_ orbital of the porphyrin is transferred to another orbital. Furthermore, the sum of the spin densities of iron and oxygen atoms is reduced from 2.0 (1.1 + 0.9) to 1.0 (0.9 + 0.1). To explain this phenomenon, we perform molecular orbital analysis and find that after the binding of the first proton, the original singly-occupied π^*^ (d_xz_-p_x_) becomes a fully-occupied d_xz_ orbital with lower energy, and the original singly-occupied a_2u_ orbital becomes empty (Scheme 2). The singly-occupied π^*^ (d_yz_-p_y_) persists during the protonation. As such, the binding of the first proton breaks one Fe‒O bond, and the corresponding Fe‒O antibonding orbital π^*^ (d_xz_-p_x_) receives one electron from the a_2u_ orbital of porphyrin and changes to d_xz_ orbital (Scheme 2). Hence, the protonation product of ^2^Por^•+^Fe^IV^=O is ^2^Por^2+^Fe^III^‒OH. In addition, energy analysis also shows that this reaction is favorable because the free energy change is − 29.70, − 24.28 and − 29.80 kcal/mol for doublet, quartet, and sextet states, respectively (Table 2).

**Table 3:** Bond length (Å) and Mayer bond order of Fe–O in Por^•+^Fe^IV^=O, Por^2+^Fe^III^–OH and Por^2+^Fe^III^–OH_2_.

Name	Length of Fe–O (Å)	Mayer bond order of Fe–O
^2^Por^•+^Fe^IV^=O	1.64	1.47
^4^Por^•+^Fe^IV^=O	1.64	1.47
^6^Por^•+^Fe^IV^=O	1.63	1.43
^2^Por^2+^Fe^III^–OH	1.83	0.82
^4^Por^2+^Fe^III^–OH	1.83	0.80
^6^Por^2+^Fe^III^–OH	1.92	0.65
^2^Por^2+^Fe^III^–OH_2_	2.30	0.23
^4^Por^2+^Fe^III^–OH_2_	2.01	0.25
^6^Por^2+^Fe^III^–OH_2_	2.27	0.26

Based on the structure of Por^2+^Fe^III^–OH which already has the first proton bond to the O atom in ^2^Por^2+^Fe^III^=O (Fig. 3), next we further calculate whether the second proton would bind to the O atom in ^2^Por^2+^Fe^III^–OH. We find the distance between the O atom and Fe atom in ^2^Por^2+^Fe^III^–OH increases from 1.83 to 2.30 Å (Table 3), and the Mayer bond order decreases from 0.82 to 0.23 (Table 3). Based on the structure (Fig. 3), it is clear that the O atom in the original ^2^Por^2+^Fe^III^–OH can be removed by the binding of a second proton, resulting in the formation of H_2_O (reaction **2** in Scheme 1). Similarly, ^4^Por^2+^Fe^III^–OH and ^6^Por^2+^Fe^III^–OH show the same trend as ^2^Por^2+^Fe^III^–OH. Meanwhile, the free energy change is − 25.07, − 28.53 and − 37.05 kcal/mol for doublet, quartet and sextet states, respectively, thereby indicating that this process can occur spontaneously (Table 2, Scheme 4).

**Scheme 3:**
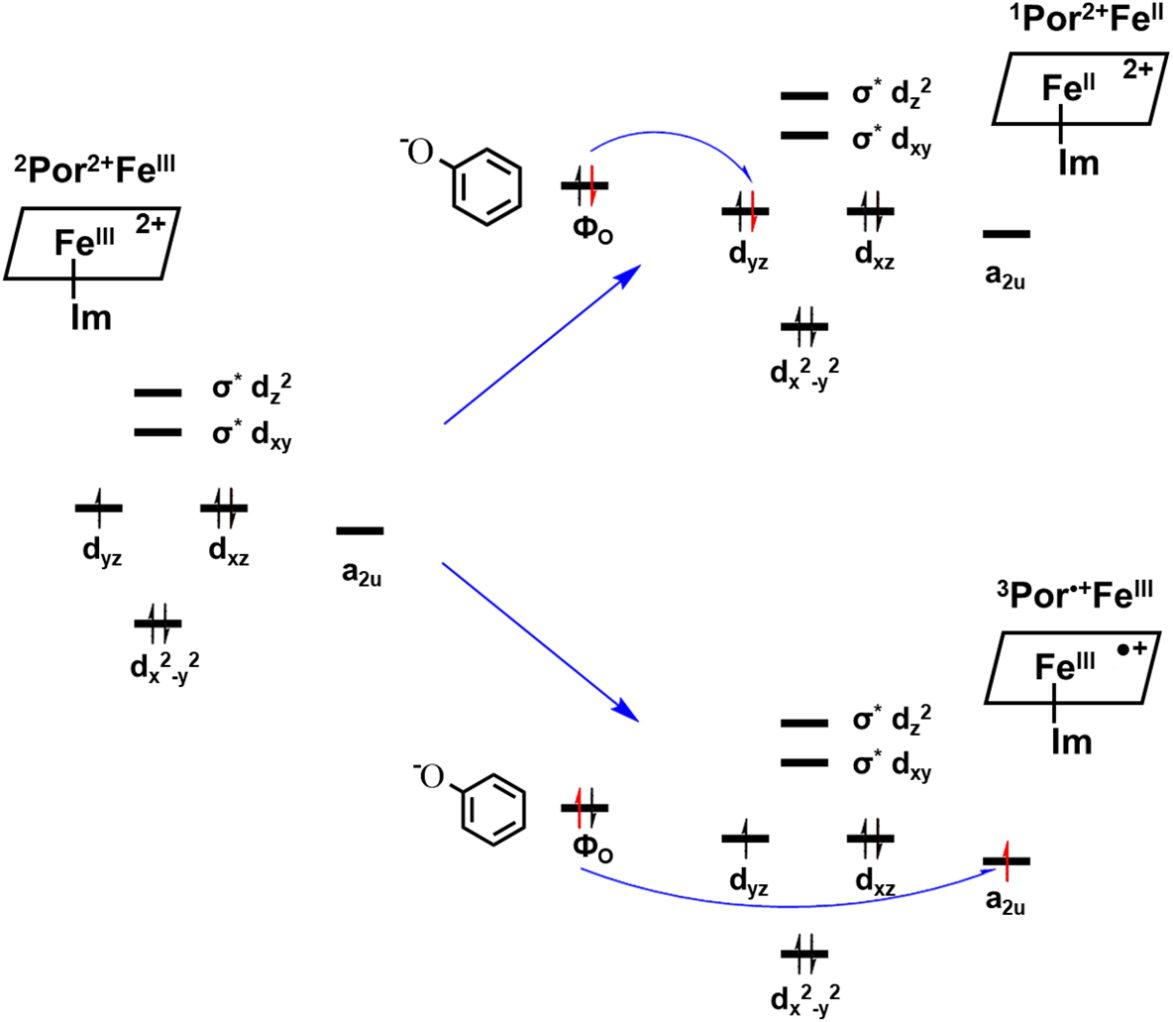
Electron transfer from phenol ion to ^2^Por^2+^Fe^III^ forming ^1^Por^2+^Fe^II^ and ^3^Por^•+^Fe^III^ during the first reduction.

**Scheme 4:**
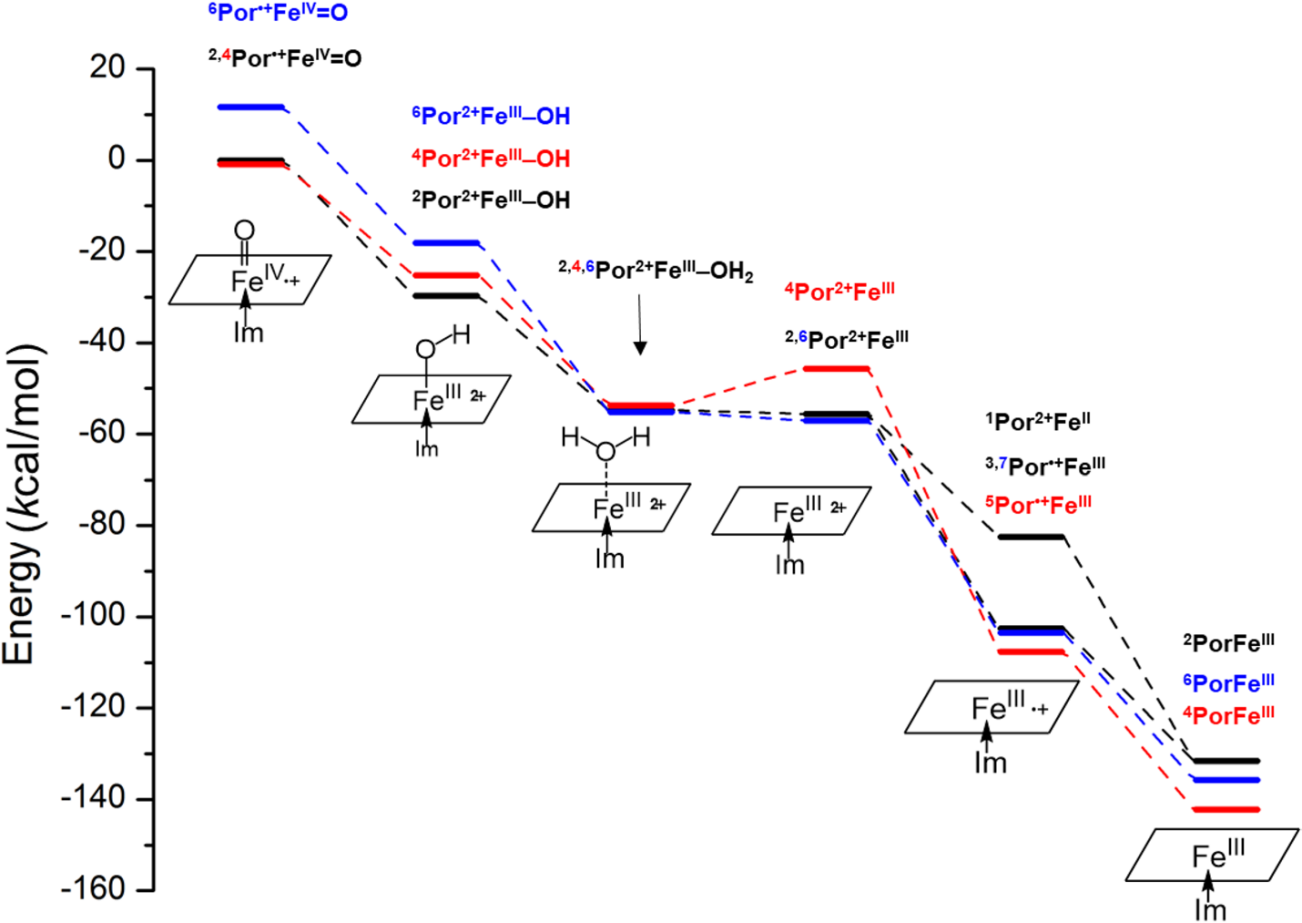
The Gibbs free energy surface of the proposed cycle.

**Figure 4:**
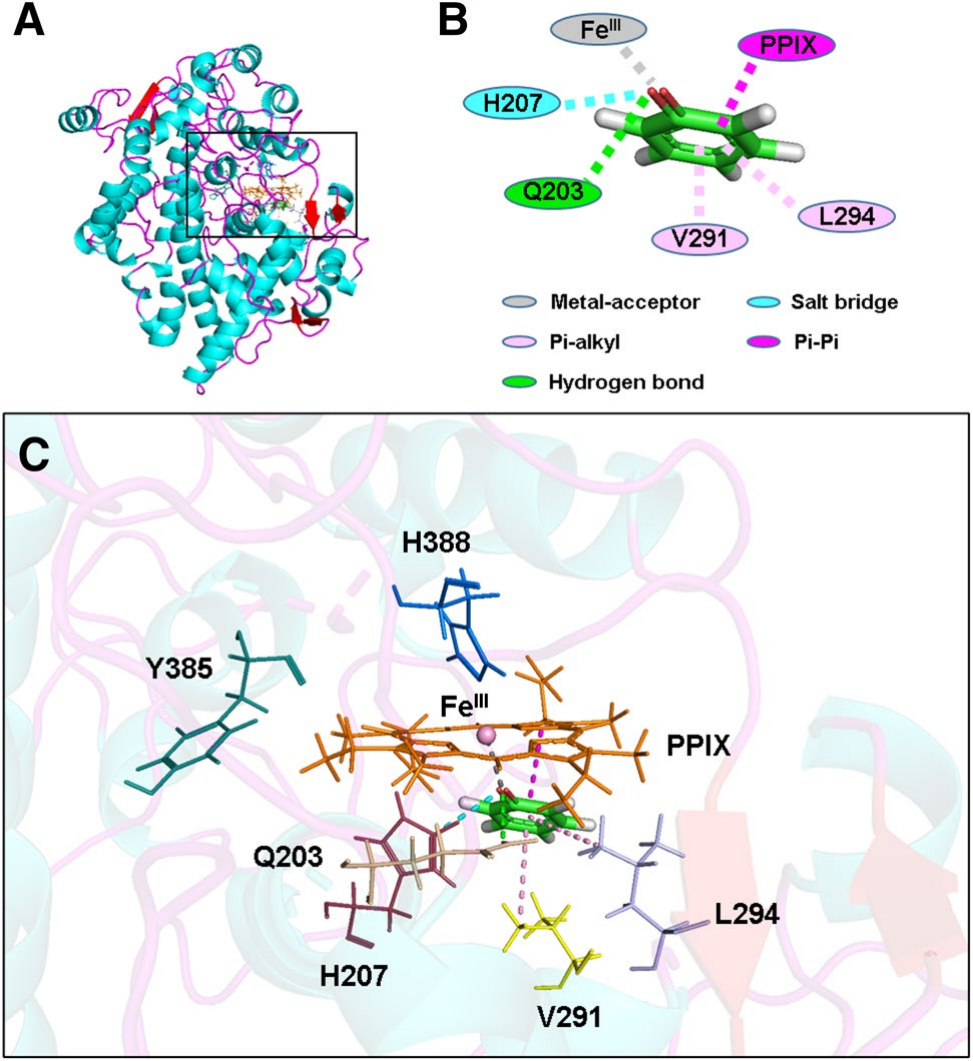
Molecular docking analysis of the binding interactions between phenol ion and amino acid residues in the peroxidase active site of human COX-2 in complex with PPIX^2+^Fe^III^ . (**A**) The structure of COX-2 in complex with phenol ion and PPIX^2+^Fe^III^. (**B**) Two-dimensional (2D) interaction diagram of the docked phenol ion and key residues in the peroxidase active site of COX-2. (**C**) The zoom-in view of the docked phenol ion inside the peroxidase site of COX-2 with PPIX^2+^Fe^III^. The protein structure is shown as solid ribbons, with different colors representing different types of the secondary structures in (**A**) and (**C**). Phenol ion is shown as sticks, with different colors representing different atomic elements. Fe^III^ is shown as sphere and colored in pink. All the nearby residues are shown in line, with PPIX^2+^ ring in orange, H388 in marine, Y385 in dark green, H207 in raspberry, V291 in yellow, Q203 in wheat, and L294 in light blue. All intermolecular interactions that facilitate the binding of phenol ion are shown in dash line, with metal-acceptor in gray, salt bridge in cyan, Pi-alkyl in pink, hydrogen bond in green, and Pi-Pi in magenta.

According to the reaction mechanism (Scheme 1), two electrons are required to restore the Por^2+^Fe^III^ to PorFe^III^. As is shown above, an ionized phenol (also called phenol ion), which is electron rich, can bind very closely to Por^2+^Fe^III^, and may readily supply an electron to it (Figs. 1 and 4). To investigate how a phenol ion donates an electron to Por^2+^Fe^III^, we calculate the spin density, NBO charge, and molecular orbital of Por^•+^Fe^III^, which is a reduction product, and compare them with those of Por^2+^Fe^III^. By mapping spin densities and NBO charges of two intermediates, we find that ^2^Por^2+^Fe^III^ becomes ^1^Por^2+^Fe^II^ or ^3^Por^•+^Fe^III^, ^4^Por^2+^Fe^III^ becomes ^5^Por^•+^Fe^III^, and ^6^Por^2+^Fe^III^ becomes ^7^Por^•+^Fe^III^, after receiving an electron. The molecular orbitals involved in the electron transfer in the case of ^2^Por^2+^Fe^III^ are depicted in Scheme 3. Compared to those of ^3^Por^•+^Fe^III^, the spin density and charge of Fe is slightly changed, while the spin density of porphyrin increases from 0.0 to 1.0, accompanied by a decrease in charge from 1.7 to 0.8 (Table 2). These results suggest that one electron is donated from phenol ion to the porphyrin ring of ^2^Por^2+^Fe^III^. Combining the above conclusion together with the analysis of the molecular orbitals of two intermediates, we propose that an electron of the phenol ion enters into the a_2u_ orbital of porphyrin. Similar electron transfers are also observed in other states of multiplicity, though the molecular orbitals involved may differ. Ultimately, the changes of Gibbs free energy demonstrate that such reduction processes are all favorable for doublet, quartet, and sextet states of Por^2+^Fe^III^ (Table 2, Scheme 4).

**Figure 5:**
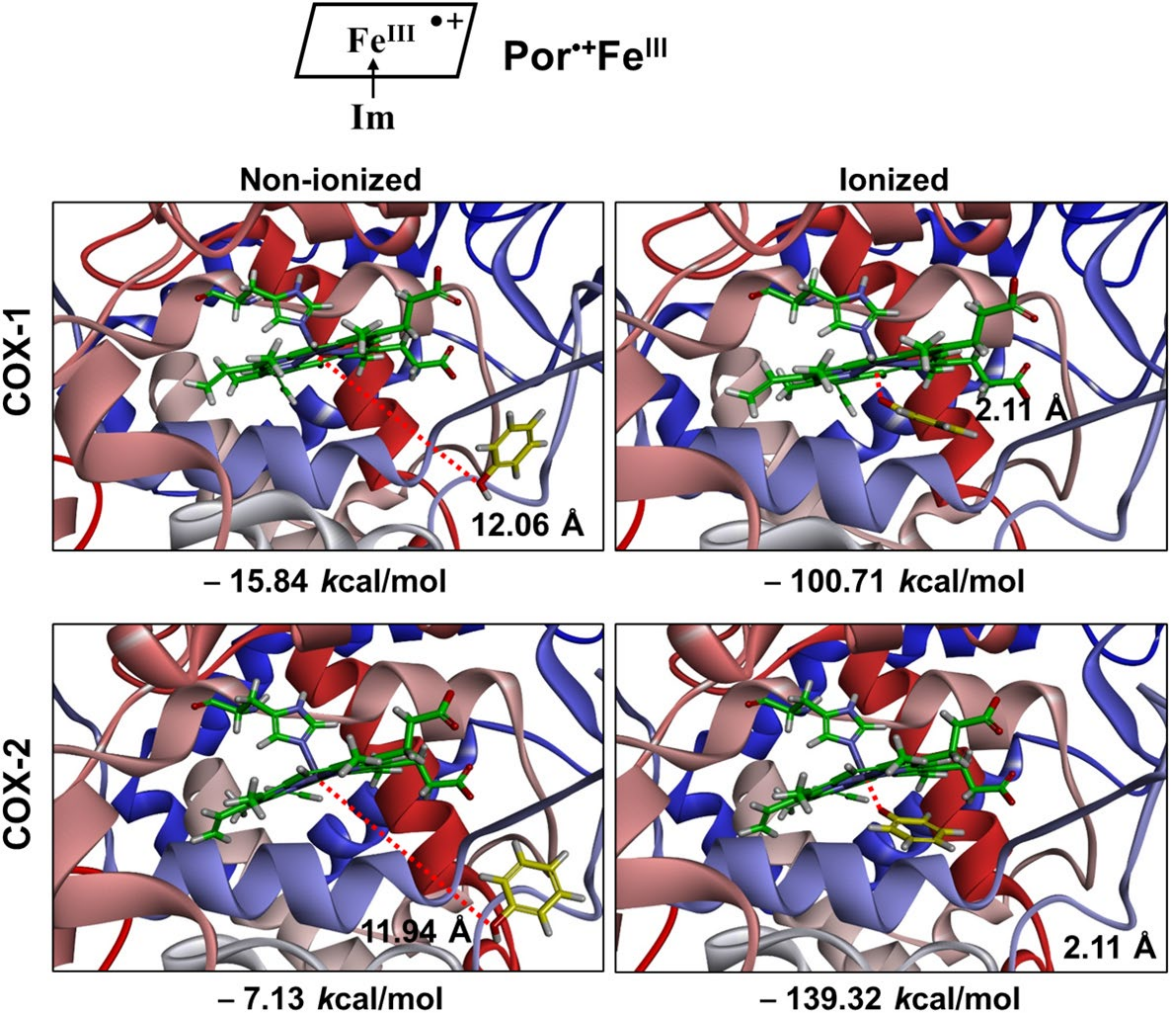
Molecular docking analysis of the binding mode of phenol in both non-ionized and ionized states inside the peroxidase active sites of human COX-1 and COX-2 in complex with PPIX^+^Fe^III^. The protein structures are shown as solid ribbons. Carbon atoms in PPIX^+^Fe^III^ are colored in green, nitrogen in blue, oxygen in red, hydrogen in white, and iron in bice. Carbon atoms in phenol are colored in yellow, oxygen in red, and hydrogen in white. In addition, the dash line corresponds to the distance between phenol’s oxygen atom and iron in PPIX^+^Fe^III^.

**Figure 6:**
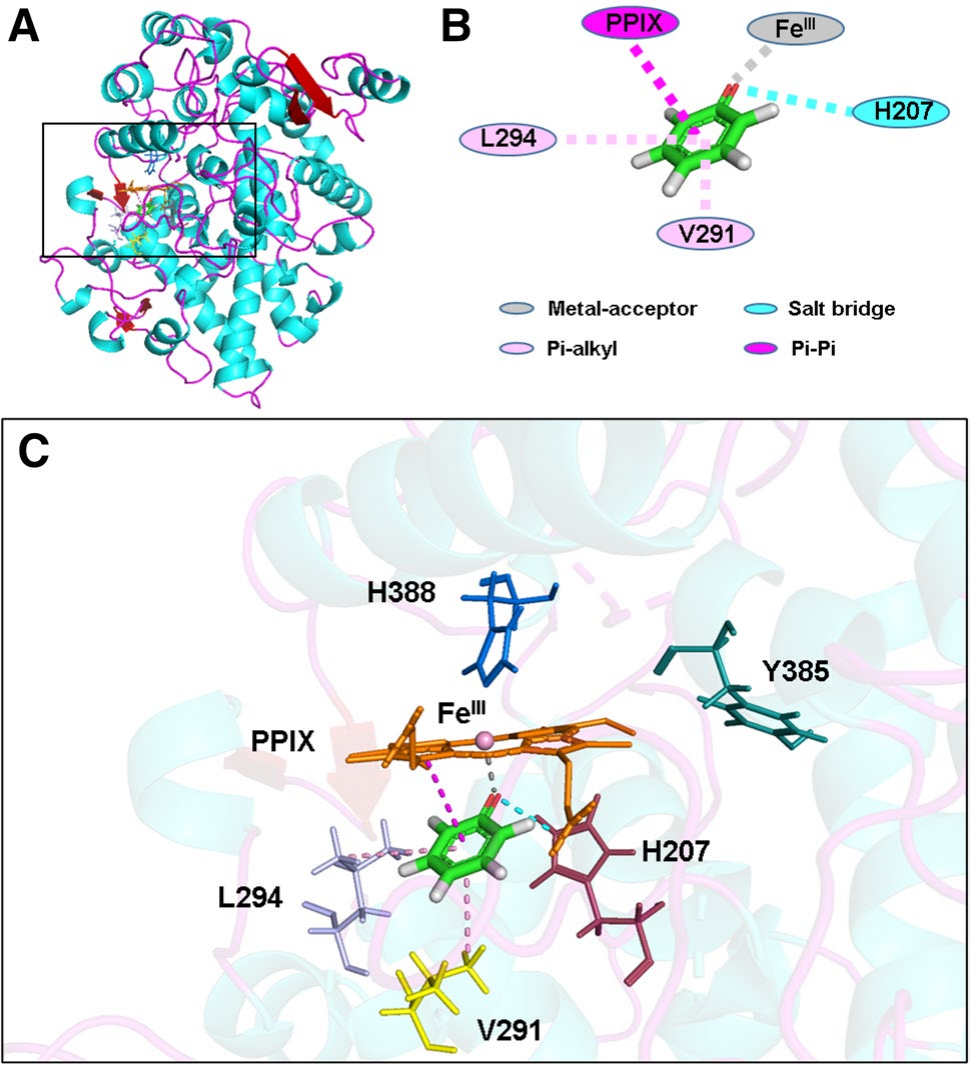
Molecular docking analysis of the binding interactions between phenol ion and amino acid residues in the peroxidase active site of human COX-2 in complex with PPIX^•+^Fe^III^ . (**A**) The structure of COX-2 in complex with phenol ion and PPIX^•+^Fe^III^. (**B**) Two-dimensional (2D) interaction diagram of the docked phenol ion and key residues in the peroxidase active site of COX-2. (**C**) The zoom-in view of docked phenol ion inside the peroxidase site of COX-2 with PPIX^•+^Fe^III^. The protein structure is shown as solid ribbons, with different colors representing different types of the secondary structures in (**A**) and (**C**). Phenol ion is shown as sticks, with different colors representing different atomic elements. Fe^III^ is shown as sphere and colored in pink. All the nearby residues are shown in line, with PPIX^•+^ ring in orange, H388 in marine, Y385 in dark green, H207 in raspberry, V291 in yellow, and L294 in light blue. All intermolecular interactions that facilitate the binding of phenol ion are shown in dash line, with metal-acceptor in gray, salt bridge in cyan, Pi-alkyl in pink, and Pi-Pi in magenta.

After the first electron is transferred from one phenol molecule to PPIX^2+^Fe^III^, Por^•+^Fe^III^ is formed in COX-1 and COX-2. Then we employ *Flexible Docking* approach again to dock the second phenol molecule in both non-ionized and ionized states into the peroxidase active site of COX-1 and COX-2 with PPIX^+^Fe^III^ (Figs. 5 and 6, Figure S4). As predicted, the results are very similar to those with PPIX^2+^Fe^III^. In the case of COX-1, the binding energy value is − 15.84 kcal/mol for non-ionized phenol and − 100.71 kcal/mol for ionized phenol, and in the case of COX-2, the binding energy value is − 7.13 kcal/mol for non-ionized phenol and − 139.32 kcal/mol for ionized phenol (Table 1). The distances between the O atom of non-ionized phenol and the Fe atom of PPIX^+^Fe^III^ are 12.06 and 11.94 Å, respectively, for COX-1 and COX-2 (Table 1). However, when phenol is ionized, the distances between its O atom and the Fe of PPIX^+^Fe^III^ are much closer, at 2.11 Å for both COX-1 and COX-2 (Table 1). It is predicted that these distances are sufficiently short for effective electron transfer to take place. Similar results are also obtained using sheep COX-1 and mouse COX-2 as target proteins (summarized in Figure S5).

When the protein surface for peroxidase active sites of COX-1 and COX-2 is modeled, it is noted that most of the amino acid residues inside the active site where phenol ion binds are neutral or basic (Figure S6). Therefore, it is speculated that the protein microenvironment in or near the active site likely would be conducive to the binding of an ionized phenol molecule. In addition, it is of note that an earlier study showed that the distal histidine (His207) may facilitate the ionization of hydroperoxide inside the peroxidase active site^43^. Similarly, it is also observed in this study that the phenol ion is very close to His207 (Figs. 4 and 6, Figure S4), making it likely that His207 may aid in stabilizing ionized phenol inside the peroxidase site. In addition, His207 may facilitate the ionization of phenol through formation of a salt bridge with phenol ion’s O^‒^ atom (Figs. 4 and 6).

The results from docking analysis suggest that ionized phenol can bind far more favorably inside the peroxidase sites of COX-1 and COX-2 than non-ionized phenol for electron donation; this result is expected as similar results were also obtained in our recent studies^38,39^. Based on known pKa value of phenol (9.98)^44^, the extent of phenol ionization around physiologically-relevant pH range (pH 7.0 – 7.4) would be very small, around 1–3‰. Despite the small percentage of ionized phenol formed in aqueous condition, its existence would make it possible to serve as a reducing cosubstrate for the COX enzymes, partly also owing to its much higher affinity for the peroxidase active sites.

**Scheme 5:**
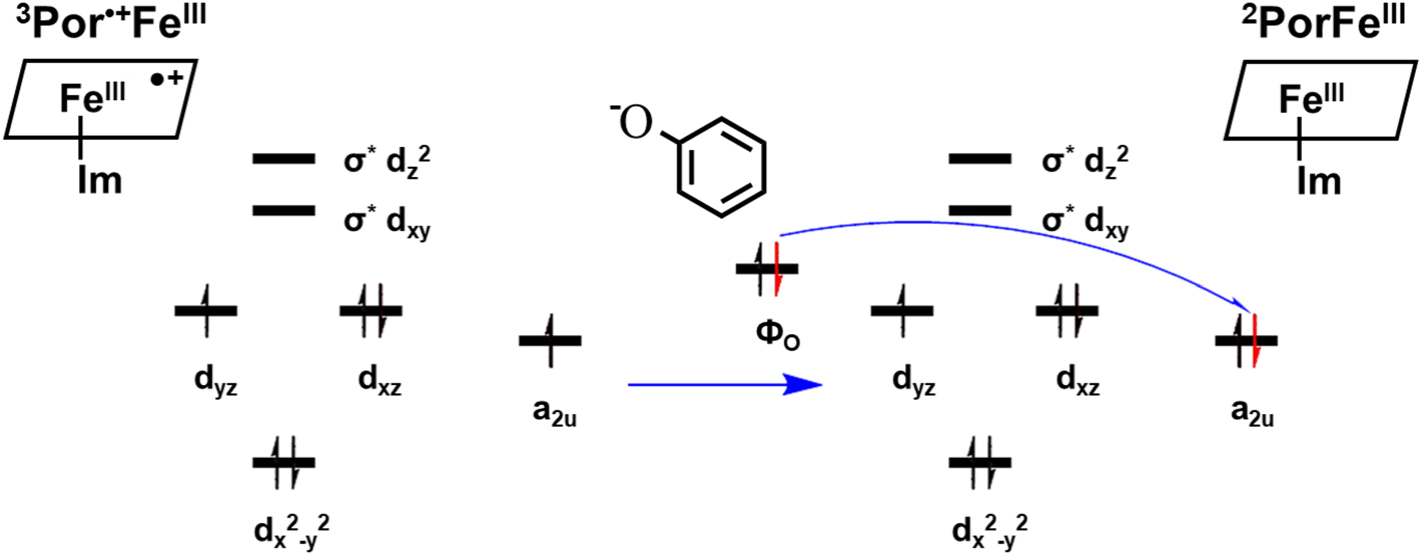
Electron transfer from phenol ion to ^3^Por^•+^Fe^III^ forming ^2^PorFe^III^ during the second reduction.

Following the above docking analysis, next we further analyze the process of Por^•+^Fe^III^ (Por^2+^Fe^II^ for singlet) reduction by the second electron via mapping spin densities and NBO charges. According to our analyses, ^1^Por^2+^Fe^II^ and ^3^Por^•+^Fe^III^ are reduced to ^2^PorFe^III^; ^5^Por^•+^Fe^III^ is reduced to ^4^PorFe^III^; ^7^Por^•+^Fe^III^ is reduced to ^6^PorFe^III^. In the case of ^3^Por^•+^Fe^III^, the spin density of porphyrin changes from 1.0 to 0.0 and its charge becomes ‒0.2 after receiving the second electron (Table 2). Therefore, we propose that the electron donated by the second phenol ion is transferred to the a_2u_ orbital of porphyrin (Scheme 5). Similar reduction processes can occur for other states of multiplicity although different molecular orbitals would participate in the electron transfer. In addition, we also calculate the change of Gibbs free energy in such processes. The second reduction is also found to be spontaneous, with free energy decrease by 29.05, 34.60 and 32.25 kcal/mol for the formation of doublet, quartet, and sextet PorFe^III^, respectively (Table 2 and Scheme 4).

Details of the potential energy profiles for the regeneration of the peroxidase activity of COX-1 and COX-2 are assembled in Scheme 4. In general, three states of multiplicity of Por^•+^Fe^IV^ can all spontaneously undergo the catalytic cycle accelerated by phenol. While the quartet and doublet states at the beginning of the reaction cycle have relatively low energy levels compared to the energy level of the sextet state, the quartet state has the lowest energy level in the last two reactions (Scheme 4). In comparison, the doublet state has the highest energy levels in the last two reactions. This information suggests that the quartet state likely is both more stable and thermodynamically more favored to proceed through the whole reaction cycle. Scheme 4 also depicts several multiplicity shifts during the whole cycle, which suggests that recombination of electrons within the orbitals may be frequent in the cycle.

**Figure 7:**
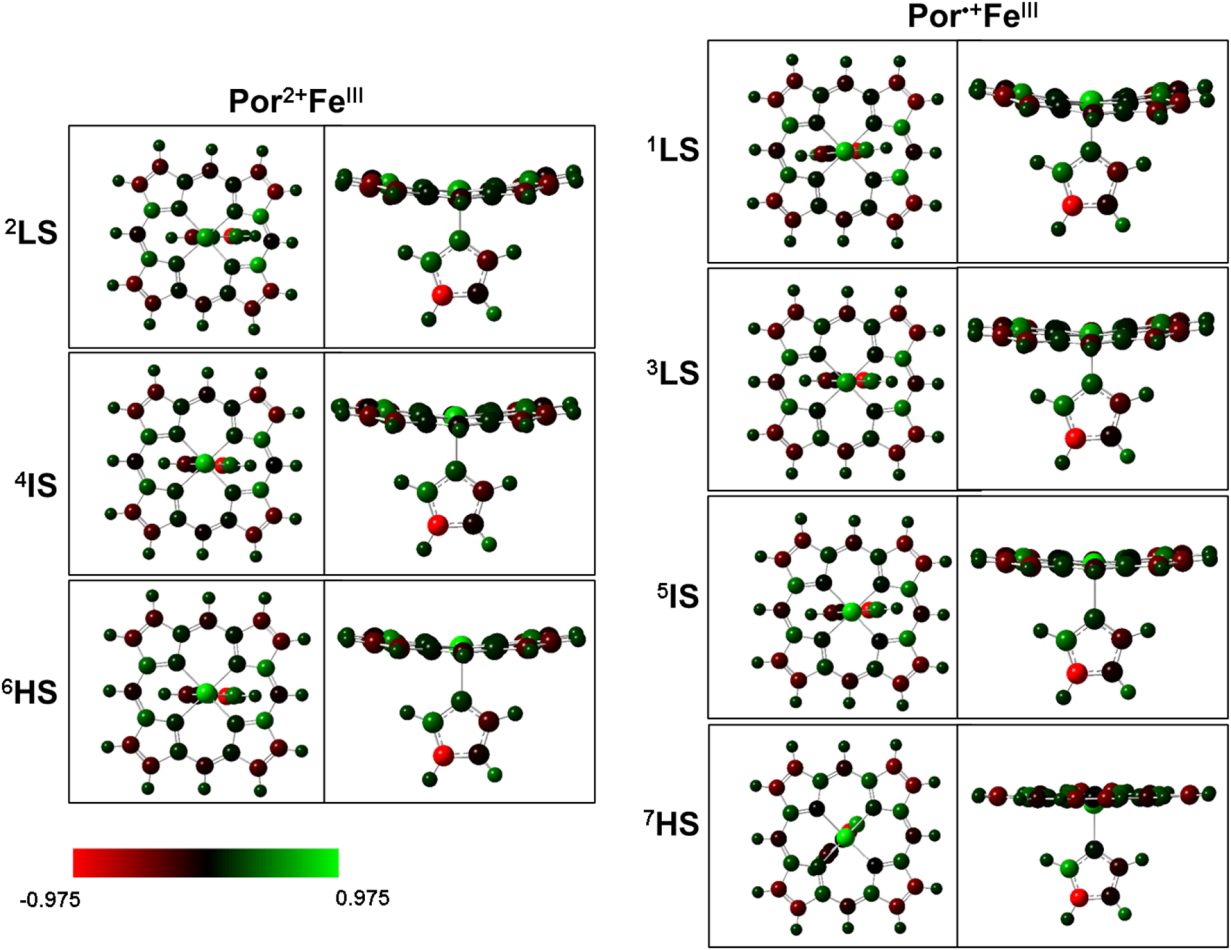
*Mulliken* charges of Por^2+^Fe^III^ and Por^•+^Fe^III^. ^1^LS singlet spin state, ^2^LS doublet spin state, ^3^LS triplet spin state, ^4^IS quartet spin state, ^5^IS quintet spin state, ^6^HS sextet spin state, ^7^IS septet spin state. The red color is for negative charges, and green for positive charges.

In our proposed cycle (Scheme 1), we hypothesize that during the reduction processes, electrons are not directly transferred from phenol ion to porphyrin. Electrons are firstly transferred to Fe (Fe^III^ in Por^2+^Fe^III^ is reduced to Fe^II^ in Por^2+^Fe^II^) and then to porphyrin (Por^2+^Fe^II^ is converted to Por^•+^Fe^III^). Therefore, it is postulated that Fe acts as a bridge of electron transfer from phenol ion to porphyrin, which is highly probable given the strong electrophilicity and polarizability of Fe and the close distance between the docked phenol ion and Fe (Table 1). In partial support of this hypothesis, Mulliken charges of atoms show that Fe ion indeed has a stronger electrophilicity compared to the porphyrin ring (Fig. 7). In addition, as shown in Scheme 4, ^1^Por^2+^Fe^II^, which is formed by electron transfer directly to Fe, displays a considerably higher energy level (about 22.46 kcal/mol higher) compared to ^3^Por^•+^Fe^III^, ^5^Por^•+^Fe^III^ and ^7^Por^•+^Fe^III^ (Table 2). This relatively large energy difference between states of multiplicity is not observed in other groups of intermediates. Therefore, it is suspected that ^1^Por^2+^Fe^II^ may serve as a reactive intermediate during the reduction and then undergo electron recombination to form ^3^Por^•+^Fe^III^, ^5^Por^•+^Fe^III^ or ^7^Por^•+^Fe^III^ (Scheme 3). Further experimental or theoretical studies are needed to test this hypothesis. It is of note that in Scheme 1, only Por^•+^Fe^IV^=O has been identified in an earlier experimental study^45^, while other theoretically-viable intermediates or transition states have not yet been identified in experimental settings thus far.

It is of note that our recent studies have shown that some of the naturally-occurring phenolic compounds such as quercetin and myricetin are high-affinity reducing substrates for the peroxidase catalytic site of COX-1 and COX-2^11,12^. Since flavonoids have similar functional groups like phenol, the results of our present study may also shed light on the mechanism of COX activation by flavonoids. The knowledge gained from this as well as earlier studies^11,12,38,39^ might aid in the design of structural analogs which can bind inside the peroxidase sites of COX-1 and COX-2 but are incapable of donating electrons for catalytic reactivation. These compounds would function as a novel type of COX inhibitors that selectively target the peroxidase sites of the enzymes and are different from clinically-used NSAIDs, which selectively target the cyclooxygenase sites.

## Conclusions

In the present study, by employing molecular docking and quantum chemistry calculation, we investigate the mechanisms by which phenol molecule activates the peroxidase catalytic cycle of COX-1 and COX-2. Computational quantum chemistry analysis shows that two protons bind sequentially to the O atom in PPIX^•+^Fe^IV^=O, resulting in oxygen removal and formation of H_2_O and PPIX^2+^Fe^III^. Following this initial reaction, two phenol ions bind in sequence inside the peroxidase sites of the enzymes, resulting in the reduction of PPIX^2+^Fe^III^ to its resting form PPIXFe^III^, with each phenol ion donating an electron. The results of our present study indicate that during the reactivation process by phenol, Fe only acts as a bridge for electron transfer, which enables the passage of two electrons from two phenol ions to Fe as a transient intermediate, and then quickly to porphyrin.

## Supplementary information

Supplementary information

## Abbreviations

COX-1 and COX-2: Cyclooxygenase 1 and 2, respectively
AA: Arachidonic acid
PG: Prostaglandin
PPIX: Protoporphyrin IX
Por: Porphyrin-imidazole complex
Im: Imidazole
