# Supplementary material for: Mechanism for the reactivation of the peroxidase activity of human cyclooxygenases: investigation using phenol as a reducing cosubstrate
Source: Sci Rep. 2020 Sep 16;10:15187. doi: 10.1038/s41598-020-71237-x (PMC7494923; doi:10.1038/s41598-020-71237-x)
Supplement: Supplementary file 1 — Supplementary information [file 41598_2020_71237_MOESM1_ESM.docx]

**Supplementary information**

**Mechanism for the Reactivation of the Peroxidase Activity of Human Cyclooxygenases: Investigation Using Phenol as a Reducing Cosubstrate^1^**

**Chengxi Yang^2^, Peng Li^2^, Xiaoli Ding, Hao Chen Sui, Shun Rao, Chia-Hsiang Hsu, Wing-Por Leung, Gui-Juan Cheng, Pan Wang^*^ and Bao Ting Zhu^*^**

Shenzhen Key Laboratory of Steroid Drug Discovery and Development (C.Y.; P.L.; X.D.; G.-J.C.; W.-P.L.; P.W., B.T.Z.), School of Life and Health Sciences (C.Y.; P.L.; G.-J.C.; P.W., B.T.Z.), and School of Science and Engineering (H.C.S.; S.R.; C.-H.H.; W.-P.L.), The Chinese University of Hong Kong, Shenzhen 518172, China; and Shenzhen Bay Laboratory, Shenzhen 518055, China (P.W.; B.T.Z.)

**Running Title: Mechanism of Cyclooxygenase Catalysis**

**FOOTNOTES:**

* Address for requests and other correspondence to: Bao Ting Zhu, School of Life and Health Sciences, The Chinese University of Hong Kong, 2001 Longxiang Road, Longgang District, Shenzhen, China 518172.

E-Mail: [**BTZhu@CUHK.edu.cn**](mailto:BTZhu@CUHK.edu.cn)

Or: Pan Wang, School of Life and Health Sciences, The Chinese University of Hong Kong, 2001 Longxiang Road, Longgang District, Shenzhen, China 518172.

E-Mail: [**wangpan@CUHK.edu.cn**](mailto:wangpan@CUHK.edu.cn)

1. This study is supported by research grants from the National Natural Science Foundation of China (NSFC No. 81473224 and No. 81630096), Shenzhen Key Laboratory of Steroidal Drug Research (No. ZDSYS20190902093417963), Shenzhen Peacock Plan (No. KQTD2016053117035204), Shenzhen Bay Laboratory (No. SZB2019062801007).

2. These two authors contributed almost equally to this study.

3. Abbreviations used: COX-1 and COX-2, cyclooxygenase 1 and 2, respectively; AA, arachidonic acid; PG, prostaglandin; PPIX, protoporphyrin IX; Por, porphorin-imidazole complex.

**Supplementary Table S1.**

***Mulliken* charge population of Por^2+^Fe^III^ and Por^•+^Fe^IV^.**

| **Atom** | **^2^Por^•+^Fe^IV^** | **^4^Por^•^+Fe^IV^** | **^6^Por^•+^Fe^IV^** | **^2^Por^2+^Fe^III^** | **^4^Por^2+^Fe^III^** | **^6^Por^2+^Fe^III^** |
| --- | --- | --- | --- | --- | --- | --- |
| **C1** | ‒0.061075 | ‒0.059179 | ‒0.113562 | ‒0.158695 | ‒0.134022 | ‒0.164697 |
| **C2** | ‒0.135382 | ‒0.075349 | ‒0.144221 | ‒0.147093 | ‒0.123860 | ‒0.164785 |
| **C3** | ‒0.036595 | ‒0.012947 | ‒0.073351 | ‒0.111007 | ‒0.151997 | ‒0.153433 |
| **C4** | ‒0.137753 | ‒0.075358 | ‒0.144154 | ‒0.143274 | ‒0.141924 | ‒0.153417 |
| **C5** | 0.103783 | 0.132101 | 0.130227 | 0.229083 | 0.136247 | 0.241821 |
| **C6** | ‒0.278154 | ‒0.294244 | ‒0.253357 | ‒0.437285 | ‒0.395377 | ‒0.427079 |
| **C7** | ‒0.356919 | ‒0.308874 | ‒0.290113 | ‒0.414063 | ‒0.361264 | ‒0.417564 |
| **C8** | 0.409807 | 0.296678 | 0.332847 | 0.346428 | 0.208364 | 0.252996 |
| **N9** | 0.131932 | 0.160335 | 0.125066 | 0.010697 | 0.060153 | ‒0.065787 |
| **C10** | 0.347016 | 0.296691 | 0.332757 | 0.287634 | 0.034177 | 0.271511 |
| **C11** | ‒0.374508 | ‒0.308871 | ‒0.290139 | ‒0.422478 | ‒0.340216 | ‒0.435600 |
| **C12** | ‒0.258135 | ‒0.294241 | ‒0.253404 | ‒0.445677 | ‒0.414305 | ‒0.435530 |
| **C13** | 0.060931 | 0.132107 | 0.130201 | 0.249400 | 0.424112 | 0.271187 |
| **N14** | 0.157041 | 0.160321 | 0.125128 | 0.038108 | ‒0.101176 | 0.038024 |
| **C15** | 0.093244 | 0.087733 | 0.076940 | 0.202410 | 0.348999 | 0.252983 |
| **C16** | ‒0.296220 | ‒0.332988 | ‒0.290974 | ‒0.452015 | ‒0.443351 | ‒0.417440 |
| **C17** | ‒0.393246 | ‒0.334812 | ‒0.316830 | ‒0.416652 | ‒0.344332 | ‒0.427116 |
| **C18** | 0.586787 | 0.401338 | 0.440599 | 0.397973 | 0.250361 | 0.241916 |
| **N19** | 0.132487 | 0.149822 | 0.118295 | ‒0.010892 | 0.057097 | ‒0.065865 |
| **C20** | 0.539663 | 0.401292 | 0.440877 | 0.359109 | 0.106885 | 0.328838 |
| **C21** | ‒0.408103 | ‒0.334795 | ‒0.316976 | ‒0.431365 | ‒0.414804 | ‒0.458389 |
| **C22** | ‒0.274097 | ‒0.332974 | ‒0.291078 | ‒0.476581 | ‒0.360370 | ‒0.458304 |
| **C23** | 0.049359 | 0.087744 | 0.076882 | 0.222069 | 0.364982 | 0.329687 |
| **N24** | 0.157157 | 0.149805 | 0.118384 | 0.015747 | ‒0.099535 | 0.033090 |
| **Fe25** | 0.543464 | 0.666764 | 0.745862 | 0.646580 | 0.735412 | 0.804970 |
| **C38** | ‒0.983594 | ‒0.970563 | ‒0.968530 | ‒1.057191 | ‒1.024811 | ‒1.031237 |
| **C39** | 0.372063 | 0.365890 | 0.373691 | 0.457698 | 0.505201 | 0.544417 |
| **N40** | 0.331915 | 0.252258 | 0.216148 | 0.217103 | 0.093789 | 0.082450 |
| **C41** | ‒0.304937 | ‒0.265149 | ‒0.272032 | ‒0.281160 | ‒0.204472 | ‒0.168293 |
| **N42** | ‒0.065253 | ‒0.083766 | ‒0.087951 | ‒0.076135 | ‒0.104754 | ‒0.086014 |

**Supplementary Table S2.**

**Total energies and Gibbs free energies (*k*cal/mol) of optimized structures.**

| **Name** | **Energy (*k*cal/mol)** | **Gibbs (*k*cal/mol)** |
| --- | --- | --- |
| **^2^Por^•+^Fe^IV^=O** | ‒1602602.73 | ‒1602418.10 |
| **^4^Por^•+^Fe^IV^=O** | ‒1602602.75 | ‒1602418.98 |
| **^6^Por^•+^Fe^IV^=O** | ‒1602589.56 | ‒1602406.43 |
| **^2^Por^2+^Fe^III^‒OH** | ‒1602875.67 | ‒1602683.68 |
| **^4^Por^2+^Fe^III^‒OH** | ‒1602870.80 | ‒1602679.14 |
| **^6^Por^2+^Fe^III^‒OH** | ‒1602860.03 | ‒1602672.12 |
| **^2^Por^2+^Fe^III^‒OH_2_** | ‒1603141.75 | ‒1602944.63 |
| **^4^Por^2+^Fe^III^‒OH_2_** | ‒1603142.54 | ‒1602943.55 |
| **^6^Por^2+^Fe^III^‒OH_2_** | ‒1603141.89 | ‒1602945.04 |
| **^2^Por^2+^Fe^III^** | ‒1555146.58 | ‒1554963.43 |
| **^4^Por^2+^Fe^III^** | ‒1555140.85 | ‒1554953.47 |
| **^6^Por^2+^Fe^III^** | ‒1555147.56 | ‒1554964.87 |
| **^1^Por^•+^Fe^III^** | ‒1555277.57 | ‒1555093.10 |
| **^3^Por^•+^Fe^III^** | ‒1555296.17 | ‒1555113.23 |
| **^5^Por^•+^Fe^III^** | ‒1555301.69 | ‒1555118.32 |
| **^7^Por^•+^Fe^III^** | ‒1555296.32 | ‒1555114.14 |
| **^2^PorFe^III^** | ‒1555430.03 | ‒1555245.14 |
| **^4^PorFe^III^** | ‒1555437.62 | ‒1555255.77 |
| **^6^PorFe^III^** | ‒1555431.24 | ‒1555249.25 |
| **Phenol** | ‒193013.31 | ‒192967.14 |
| **[Phe]-** | ‒192711.71 | ‒192673.75 |
| **C_6_H_6_O•** | ‒192608.69 | ‒192570.89 |
| **H_2_O** | ‒47983.72 | ‒47982.03 |
| **H_3_O^+^** | ‒48227.90 | ‒48217.91 |
| **H^+^** | ‒103.27 | ‒109.85 |

**Supplementary Scheme S1.**

**Electronic structure and molecular orbitals of ^4^Por^•+^Fe^IV^=O and ^2^Por^•+^Fe^IV^=O.**

**
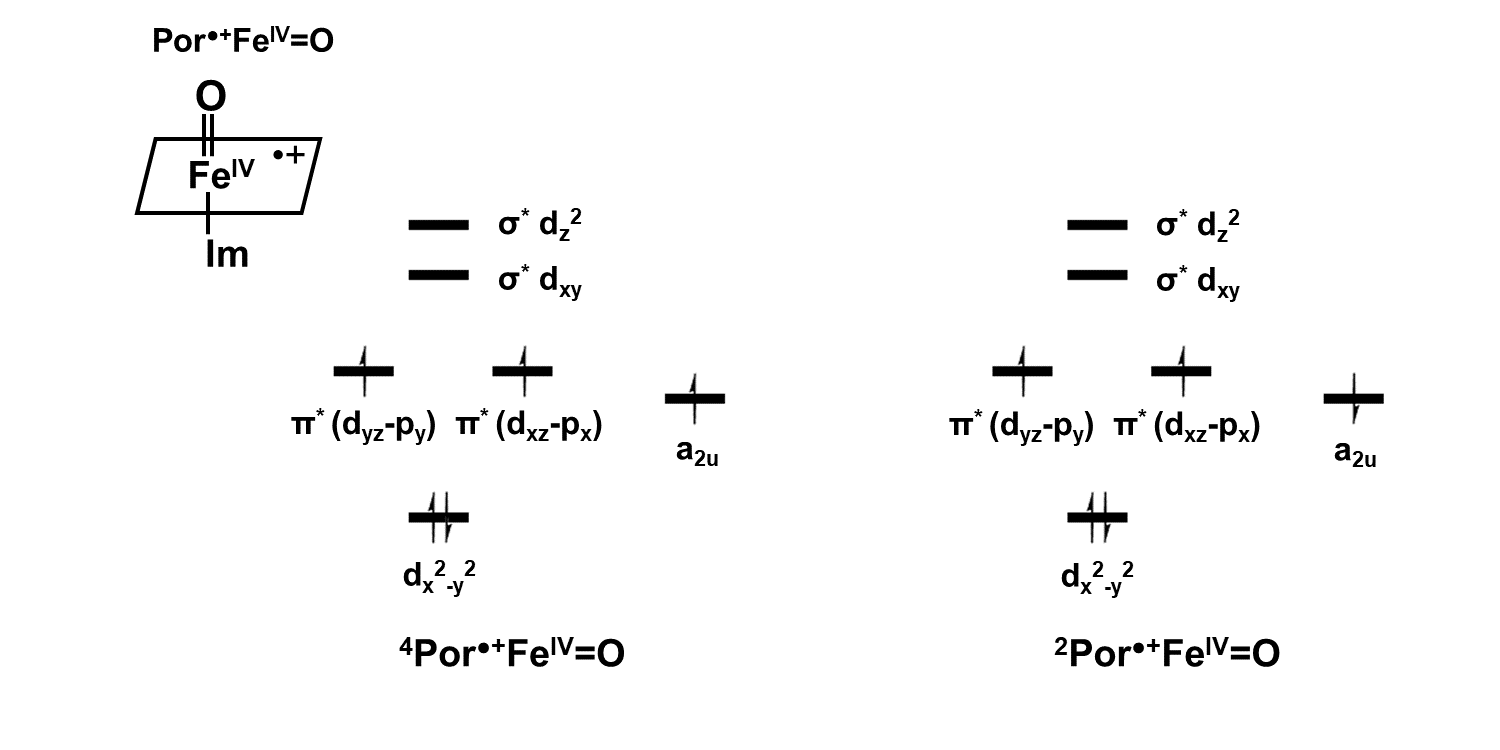
**

**Supplementary Figure S1.**

**“Self-docking” result of celecoxib that is docked back into the mouse COX-2 structure. A.** Comparison of the docked pose of celecoxib (in green) with the experimentally-determined *x*-ray structure of celecoxib (in orange). **B.** The zoom-in view of the docked (in green) and crystal structure (in orange) of celecoxib.

**
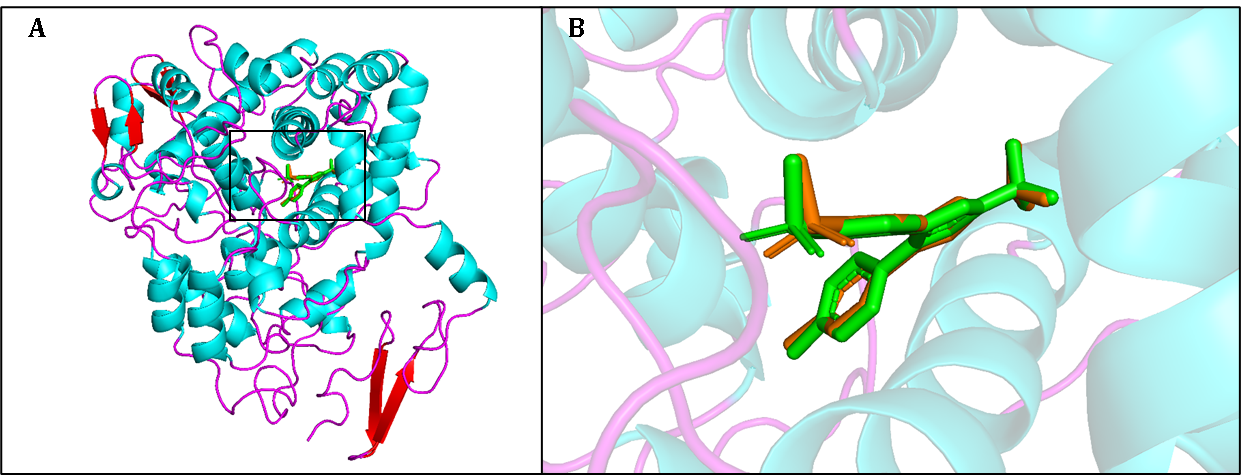
**

**Supplementary Figure S2.**

**Molecular docking analysis of phenol in both non-ionized and ionized states inside the peroxidase active sites of mouse COX-1 and sheep COX-2 in complex with PPIX^+^Fe^IV^=O and PPIX^2+^Fe^III^.**

**
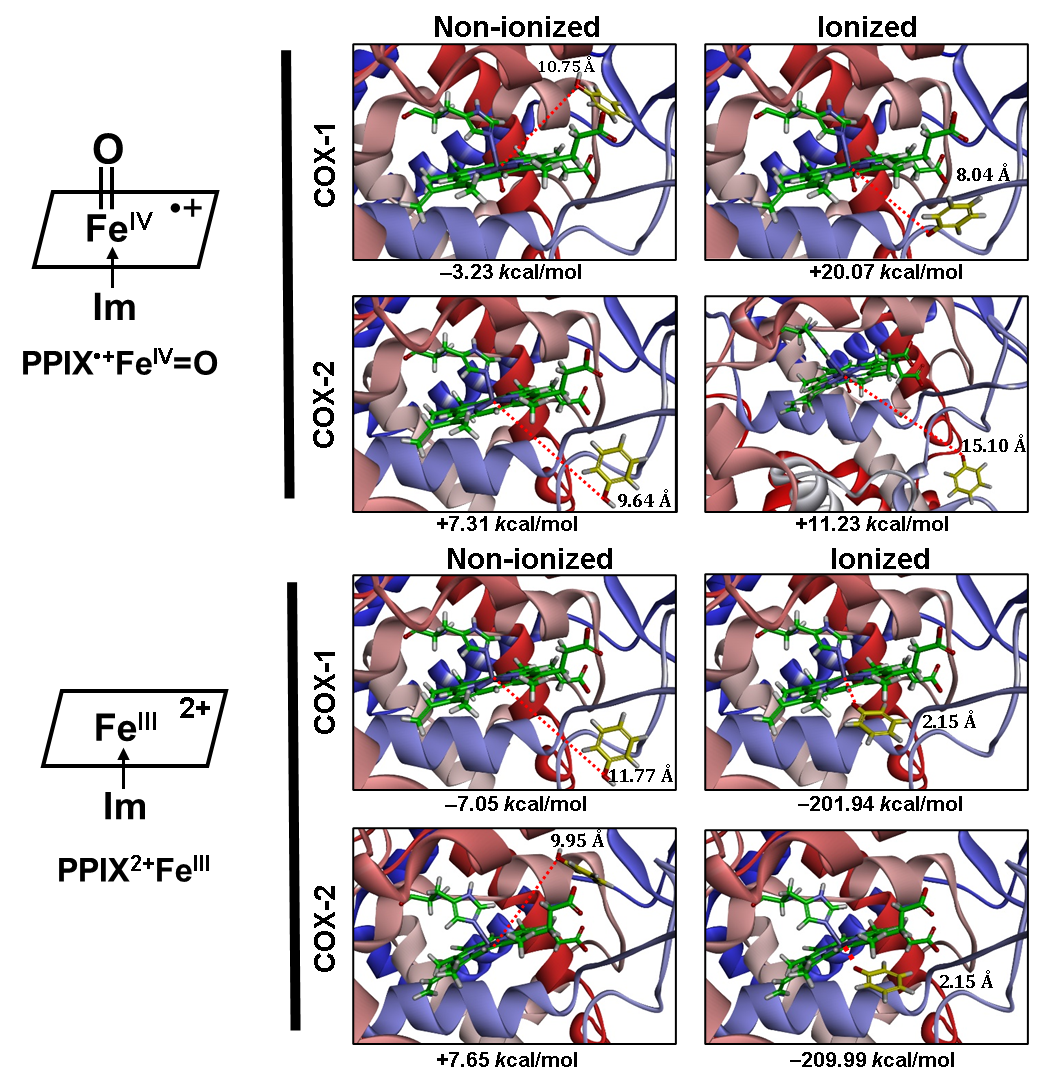
**

**Supplementary Figure S3.**

**Electronic structure and molecular orbitals of ^2^Por^•+^Fe^IV^=O and ^4^Por^•+^Fe^IV^=O.**

**
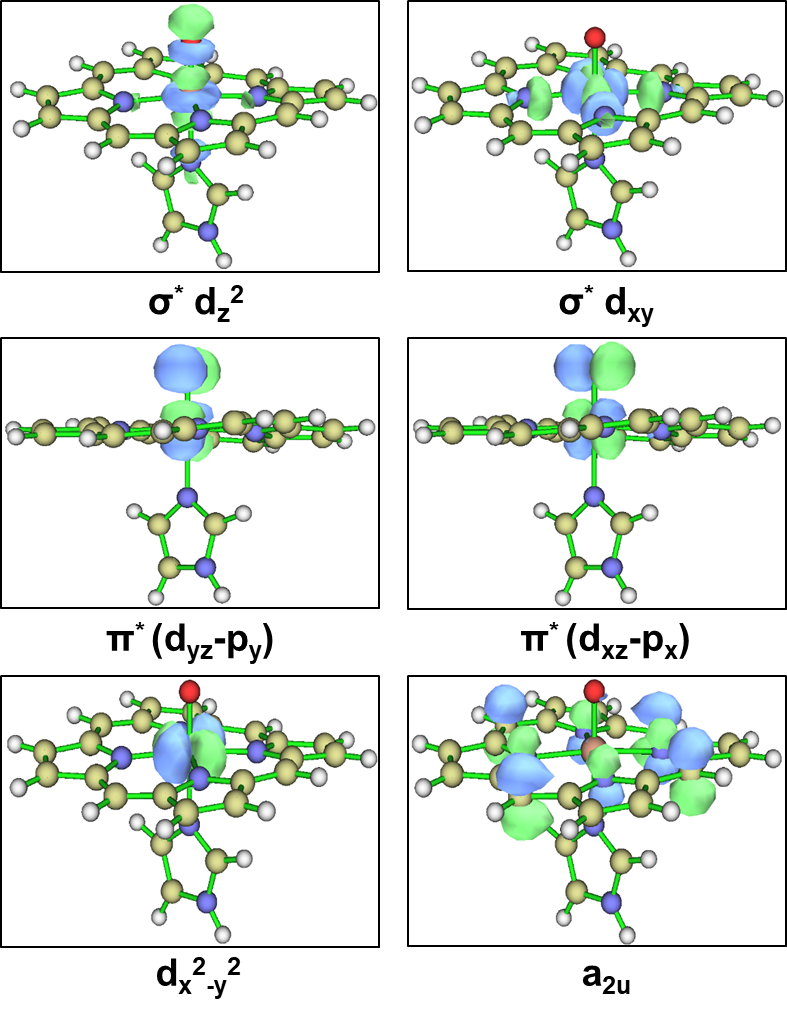
**

**Supplementary Figure S4.**

**Molecular docking analysis of the binding interactions between phenol ion and amino acid residues in the peroxidase active site of human COX-2 in complex with PPIX^•+^Fe^III^.**

**
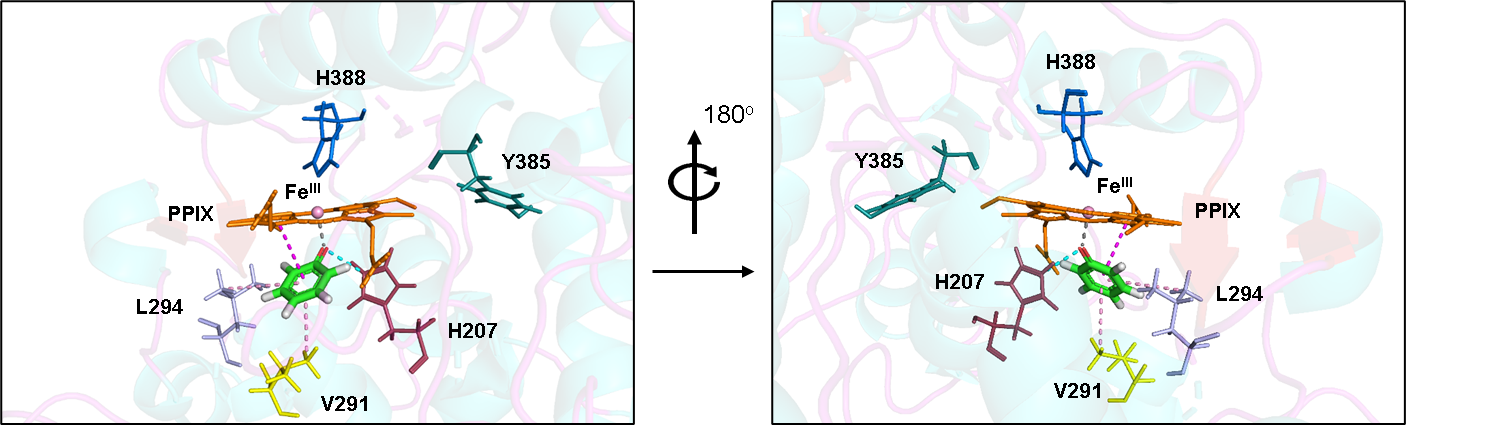
**

**Supplementary Figure S5.**

**Molecular docking analysis of the binding mode of phenol in both non-ionized and ionized states inside the peroxidase active site of mouse COX-1 and sheep COX-2 in complex with PPIX^+^Fe^III^.**

**
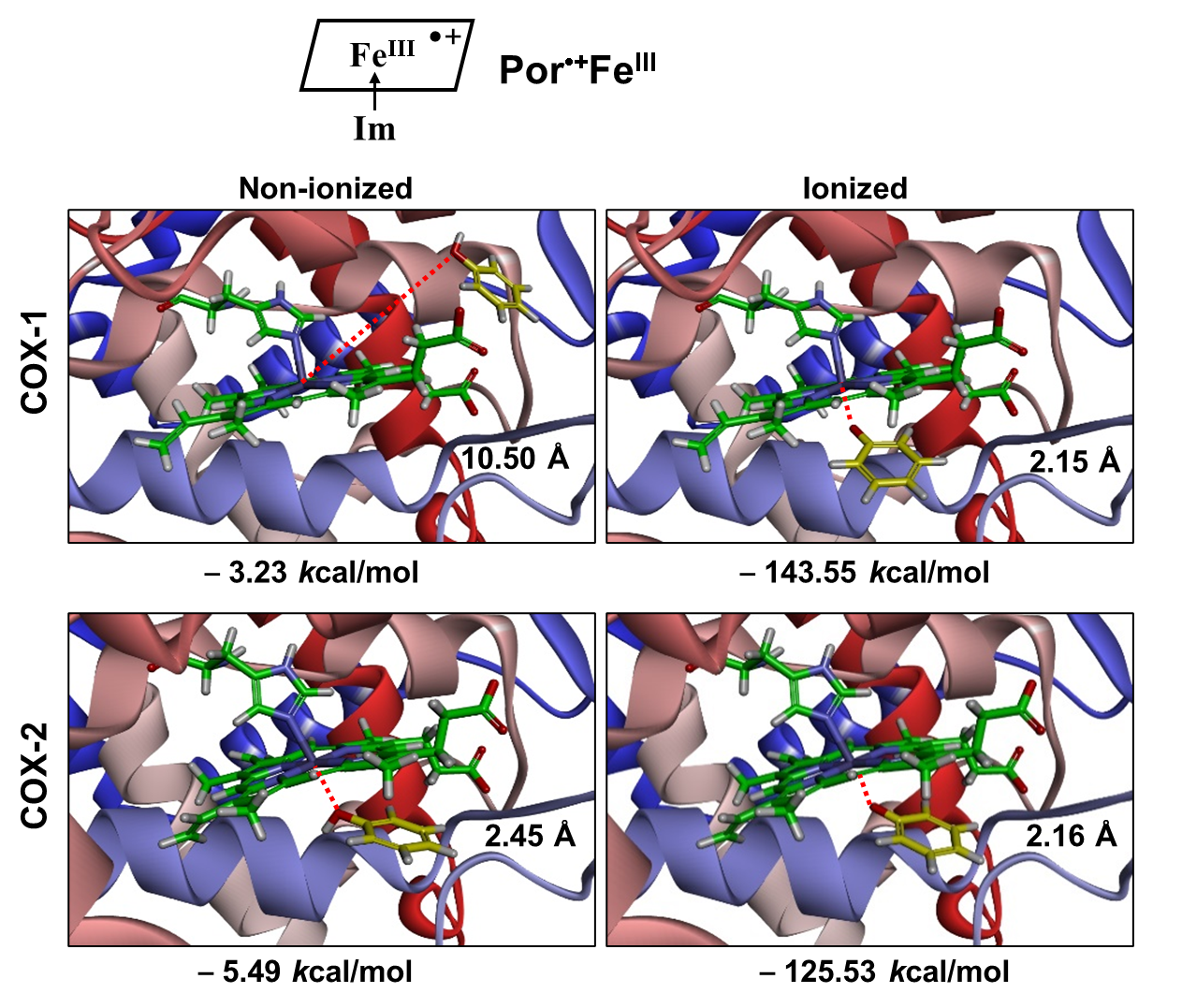
**

**Supplementary Figure S6.**

**Protein surface analysis of the acidic, neutral and basic properties of the peroxidase sites of human COX-1 and COX-2. Phenol and heme structures are shown as sticks.**

**
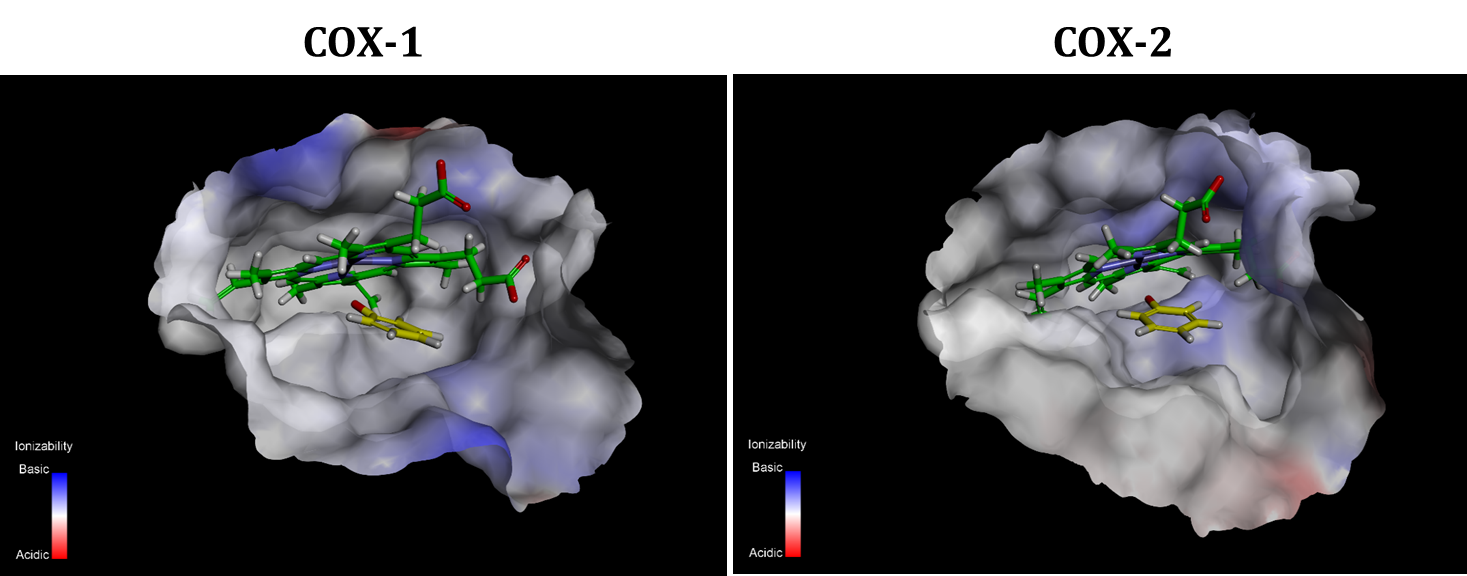
**
